# Individual differences and self-regulatory factors are credible determinants of physiotherapy student performance on clinical placement: Insights from a measurement burst design study

**DOI:** 10.1007/s10459-025-10453-4

**Published:** 2025-08-04

**Authors:** Alan Reubenson, Hugh Riddell, Margo L. Brewer, Leo Ng, Daniel F. Gucciardi

**Affiliations:** 1 Curtin School of Allied Health, Curtin University, Perth, Australia; 2 Curtin School of Population Health, Curtin University, Perth, Australia; 3 Department of Nursing and Allied Health, School of Health Sciences, Swinburne University of Technology, Melbourne, Australia

**Keywords:** Psychosocial, Determinants, Clinical performance, Measurement burst

## Abstract

Clinical placements are integral to any health professional education program, allowing students to integrate theoretical knowledge and skills acquired in campus-based learning into authentic, real-world healthcare settings. Limited empirical knowledge exists about the determinants of clinical performance. Contemporary thought is that clinical performance is determined by multiple cognitive, environmental, psychological and social factors. Accordingly, we examined which individual differences and self-regulatory factors are credible determinants of undergraduate physiotherapy student performance within clinical placements. Utilising a measurement burst design, we captured weekly self-reported survey data amongst two cohorts (2019 and 2020) of final year, undergraduate students as they progressed through four 5-week physiotherapy clinical placements. In total, 97 of the 181 consenting students met the data inclusion criteria by completing more than three surveys in a minimum of two clinical placements. For these students, we collated their clinical placement results and utilised multilevel structural equation modelling to examine intra-individual dynamics of diverse psychosocial factors and their association with clinical performance data. Study findings supported stable individual differences, contextual experiences, and self-regulatory factors as meaningful determinants of physiotherapy entry-level performance. Knowledge of salient psychosocial determinants associated with clinical performance can inform curriculum design. Doing so includes practical strategies for early identification of students who may struggle in clinical placement settings, and ways to foster salient student skills and abilities before and during placements to optimise clinical placement performance outcomes.

**Supplementary Information:**

The online version contains supplementary material available at 10.1007/s10459-025-10453-4.

## Introduction

Clinical placements are essential to health professional training, complementing campus-based activities by transitioning learning to authentic, real-world settings where students can develop and refine professional competencies (Patton et al., 2013, 2018; Penman et al., 2023). Irrespective of previous academic performance (Horwitz et al., 2023; Milgate et al., 2024; Paynter et al., 2022), many students struggle in varying degrees to navigate this transition because they encounter diverse events that challenge their capacities to perform well (Almhdawi et al., 2018; Delany et al., 2015; Gallasch et al., 2022; Kurunsaari et al., 2022). With the high costs associated with educating health professionals (e.g., ~ USD173,000 for physiotherapists and ~ USD450,000 for medicine (Segal et al., 2017)), and the high cost of under-performance (i.e., ~ USD9,000 for failing a 5-week physiotherapy placement (Foo et al., 2017)), it is imperative we advance our understanding of factors that might augment or diminish student performance within clinical placements. We contribute to this gap in knowledge by studying diverse psychosocial determinants across contextual and temporal dynamics among physiotherapy students undertaking multiple clinical placements. This knowledge can inform conceptual models of clinical education or work-integrated learning dynamics, practical strategies for designing curricula, and targeted support structures to optimise student outcomes and improve graduate work-readiness.

The contextual demands of academic and clinical education differ greatly. Academically, learning and performance typically occurs on-campus and/or online; encompasses activities that take place in lectures, tutorials, and workshops; and is primarily evaluated via written (e.g., assignments, examinations) and/or practical (e.g., viva voces, Objective Structured Clinical Examination (OSCE)) assessments. Academic performance, therefore, reflects a quantitative approximation of students’ degree of mastery of cognitive knowledge and/or practical skills salient to the discipline that are acquired and assessed via controlled, formal processes (e.g., exams, test) within tertiary educational settings. In contrast, clinical performance encapsulates quantitative approximations via observations of practice within the context of in/direct patient care regarding students’ degree of mastery in the application of salient knowledge, skills, and procedures in dynamic, real-world settings alongside or supervised by health professionals. Assessments of clinical performance typically consider numerous competencies that span cognitive (e.g., critical thinking, problem-solving), technical (e.g., diagnostic procedures, manual therapy interventions), interpersonal (e.g., verbal and written communication, undertaking a subjective examination, interprofessional collaboration), evidence-based practice, and professional (e.g., ethics, responding favourably to feedback) domains (Epstein & Hundert, 2002; Institute of Medicine Committee on the Health Professions Education, 2003; World Physiotherapy, 2021). Thus, learning in academic settings is foundational and a prerequisite to the development of clinical performance capabilities, yet the demands of, and associated resources required for, successful clinical practice vary greatly from those required in academic settings (Cooper et al., 2010; Peters et al., 2017).

Relative to research on academic performance, scholarly work on the correlates or determinants of clinical performance is limited to a small number of studies across medicine (Ferguson et al., 2002; Hamdy et al., 2006; Kim et al., 2016; Stegers-Jager et al., 2015), nursing (Mergal et al., 2018; Pitt et al., 2012), occupational therapy (Horwitz et al., 2023; Reynolds et al., 2021; Wang et al., 2023), speech-language pathology (Johnson et al., 2021; Reynolds et al., 2021), and physiotherapy (Horwitz et al., 2023; Reynolds et al., 2021). General factors including cognitive ability (e.g., pre-admission tests) and past academic achievement (e.g., high school GPA) are typically less meaningful determinants of clinical performance than specific factors such as preclinical GPA (Ferguson et al., 2002; Hamdy et al., 2006; Horwitz et al., 2023; Kim et al., 2016; Stegers-Jager et al., 2015) or earlier clinical placement performance (Johnson et al., 2021). Relatedly, via a systematic review of 18 observational studies primarily within the medical profession, OSCEs - a proximal measure of technical skills and communication - explained ~ 1-40% of the variance in student clinical performance (Terry et al., 2017). Psychosocial determinants of clinical performance remain under-explored, with initial results suggesting factors such as emotional intelligence (Mergal et al., 2018; Wang et al., 2023), self-efficacy (Johnson et al., 2021; Mergal et al., 2018; Opacic, 2003; Pasupathy & Bogschutz, 2013; Reynolds et al., 2021; Rice, 2015), resilience (Brown et al., 2020, 2022), grit (Calo et al., 2022; Terry & Peck, 2020), conscientiousness (Hojat et al., 2013; Milne et al., 2019; Paynter et al., 2023), learning style and approach (Landa-Gonzalez et al., 2015; Paynter et al., 2023), and social support (Mergal et al., 2018; Paynter et al., 2023) may contribute positively to clinical performance. The interplay of stress with clinical performance remains unclear, with studies in simulated and real-world settings providing contrasting results, highlighting the underlying complexity of stress and other psychosocial dimensions (Al-Ghareeb et al., 2017; Mergal et al., 2018; Reynolds et al., 2021). In professions like physiotherapy where clinical and/or practical competencies are essential for entry-level graduates, admission into most, if not all, programs is heavily reliant on past academic achievement, yet the available evidence indicates that academic performance alone provides an incomplete picture of key determinants of clinical performance.

Clinical placements are complex and dynamic learning and development spaces in which students’ capabilities and circumstances interact with contextual workplace elements, supervisor characteristics, and patient and other health professional encounters (Patton et al., 2018). The Process-Person-Context-Time (PPCT) socioecological model (Dickson & Darcy, 2021) provides an integrative framework upon which to consider these multiple interactions. Essentially, within health professional courses like physiotherapy, students undertake several independent clinical placements (e.g., cardiorespiratory, musculoskeletal) across diverse placement settings (public/private, acute/rehabilitation) under the supervision of qualified health professionals. A key feature of the PPCT framework, which is most salient to the context of determinants of clinical performance, is the temporal sequencing and longitudinal nature of person-context interactions. In essence, none of the individual PPCT elements are static. *Processes* and *contexts* vary within placements (e.g., encountering un/familiar patient presentations) and between placements (e.g., different locations, supervisors). It is also erroneous to view the *person* (e.g., students, patients) as a static dimension within a broader, changing ecosystem. Doing so overlooks the learning and development that occurs over *time*. One’s resources (e.g., social support) and capabilities (e.g., resilience) also fluctuate over time and are sometimes contextually-bound (Hobfoll et al., 2018; Zhang et al., 2023). For example, students may be more confident in slower-paced rehabilitation settings than they are in faster-paced acute care settings (Phan et al., 2023). Thus, a holistic understanding of key determinants of clinical performance demands consideration of individual student factors salient to the unique pressures of physiotherapy clinical placements.

People strive to acquire, maintain, utilise, and safeguard resources they value and bring with them to every encounter or experience (Hobfoll, 1989; Hobfoll et al., 2018). From an individual differences standpoint, resources can be broadly considered as knowledge, skills, abilities, and other characteristics (KSAOs) that influence how students engage with learning and practice during clinical placements (Ployhart et al., 2014). Of these four categories, researchers have paid the most attention to knowledge via proxies of cognitive ability (e.g., GPA), with the available evidence supporting small-to-moderate positive associations with physiotherapy clinical performance (Howard & Jerosch-Herold, 2000; Paynter et al., 2022; Terry et al., 2020; Tidd & Conine, 1974). The evidence on the association between skills with physiotherapy clinical performance among the only three studies to date is mixed, with observations of positive associations between OSCE scores (Terry et al., 2020) or critical self-reflection skills (Brooks et al., 2017) and clinical performance, and the absence of any meaningful association with OSCE scores (Wessel et al., 2003). Findings regarding the association between students’ abilities with physiotherapy clinical performance is also mixed, with efforts limited to generic factors including emotional intelligence (Larin & Wessel, 2015; Lewis, 2010, 2011), moral reasoning (Larin & Wessel, 2015; Sisola, 2000), grit (Calo et al., 2022), resilience (Calo et al., 2022), self-efficacy (Reynolds et al., 2021), mindset (Calo et al., 2022) and other characteristics, such as behavioural styles (Milne et al., 2019), and personality traits (Paynter et al., 2023). Empirical knowledge of key determinants of clinical performance is limited and therefore insufficient for informing targeted interventions, processes, or policies. Efforts to address this evidence gap must consider diverse factors in ways that align with the complex and dynamic nature of clinical performance.

Compounding the limited breadth and depth of potentially important psychosocial determinants of clinical performance within existing research is the mismatch between the nature of the problem and the study designs employed to uncover empirical knowledge. Clinical performance, particularly within the context of tertiary education where students are assessed on multiple occasions regarding their readiness to enter the workforce, is best conceptualised as a within-person or intra-individual concept, yet what we know draws primarily from between-person or inter-individual evidence. Methodologically, the modus operandi among existing clinical performance research is one that relies on cross-sectional snapshots of determinants and clinical performance outcomes. The focus on mean levels of clinical performance and key determinants also overlooks within-person dynamics; for example, many psychosocial factors are state-like in nature and likely develop or fluctuate in the short-term (e.g. availability of support from peers over a week) and long-term (e.g. self-efficacy beliefs over the duration of a tertiary health science course). Additionally, the assumption of homogeneity of variance required for most statistical examinations of mean levels presumes student performance is largely invariable across different placement settings (e.g., acute, rehabilitation) and across time. This incompatibility between concept and method is problematic because it assumes evidence at the population level (between-person) generalises to individuals (within-person), which often is not the case (Fisher et al., 2018). Repeated assessments of psychosocial and contextual factors within and across multiple placements and time, as well as disentangling averages from within-person variability of these concepts, would provide a nuanced overview of clinical performance.

Against this conceptual and empirical backdrop, we aimed to examine the within-person and between-person associations between diverse psychosocial factors and clinical performance among physiotherapy students. The overarching research question was: which individual differences and self-regulatory factors are credible determinants of undergraduate physiotherapy student performance within clinical placements? Our selection of psychosocial and contextual factors (see Table 1 for an overview) aimed to strike a balance between breadth and depth regarding potentially salient factors, as well as pragmatic considerations (e.g., participant burden). Given the exploratory nature of this work, we sought to generate empirically-informed inferences regarding psychosocial determinants which are credibly associated with student clinical performance.

**Table 1:** Overview of study variables and their operationalisation via measurement scales

Construct	Description and item numbers	Item Examples	Rating Scale and Descriptive Anchors	Scoring Protocol
**Level 1 (between-persons) Variables**				
Academic Self-Efficacy	Using established guidelines (Bandura, 2006), we created a 16-item study-specific scale to assess students’ belief in their ability to perform well on a range of coursework learning competencies. Of 42 undergraduate physiotherapy teaching staff at Curtin University, 24 (57%) responded to an invitation to complete a survey requesting their perceptions of key skills required by students to be successful in the academic (not clinical) components of the course. The lead author thematically analysed the qualitative responses, with support from [DG and LN]. The final item pool consisted of behavioural items (8), cognitive items (6), and emotional items (2).	Stem: On a scale of 0–10 rate your degree of confidence in… breaking down complex information into simple, easy to remember concepts. retaining large amounts of information. managing your emotional state (e.g. stress, anxiety) effectively during practical examinations.	11-point scale 0 (no confidence at all) 5 (moderate confidence) 10 (complete confidence)	Average of all 16 items.
Perfectionism	We utilised the 8-item Frost Multidimensional Perfectionism Scale-Brief (FMPS-B) to assess students’ perfectionistic tendencies in terms of excessively high achievement strivings (4-items) and overly critical self-evaluations (4-items) (Burgess et al., 2016).	Evaluative concerns: The fewer mistakes I make, the more people will like me. If I fail at work/school, I am a failure as a person	5-point scale 1 (Strongly disagree) 2 (Disagree) 3 (Neither agree nor disagree) 4 (Agree) 5 (Strongly agree)	Average of all items for each subscale.
		Striving: I set higher goals for myself than most people. Other people seem to accept lower standards from themselves than I do		
Lifetime Adversity	We utilised an inventory of lifetime adversity events (Lines et al., 2020) that captures categories of own illness or injury, loved one’s illness or injury, violence, bereavement, social/environmental stress, relationship stress, threat or harassment, and others’ death or injury. Students were asked to report if they’ve experienced an event in their lifetime and, if so, how many times.	Number of events experienced: Stem: Please indicate if you have ever in your life experienced any of the following events… Serious physical attack or assault. Domestic violence. Death of a loved one (e.g. parent, sibling).	No Yes Not Applicable	We calculated a summed score to reflect (1) total number of different adversities experienced and (2) their frequency of occurrence.
		Frequency: Stem: Please indicate how many times you have experienced the event	6-point scale (1–6 or more) and not applicable	
Persistence	We utilised the 4-item Persistence subscale from the Motivation Engagement Scale– University/College (MES-UC) to measure the extent to which students sustain engagement towards their studies (Liem & Martin, 2012).	If I can’t understand my university work at first, I keep going over it until I do. When I’m taught something that doesn’t make sense, I spend time to try and understand it.	7-point scale 1 (Strongly disagree) 2 (Disagree) 3 (Somewhat disagree) 4 (Neither agree nor disagree) 5 (Somewhat agree) 6 (Agree) 7 (Strongly agree)	Average of all 4 items.
Big-5 Personality trait– Extraversion	We utilised the short 15-item Big Five Inventory (BFI-S) to assess the personality traits of extraversion, agreeableness, conscientiousness, neuroticism, and openness (Hahn et al., 2012; Lang et al., 2011). Conceptual definitions (John et al., 2008, p.138): “*Extraversion* implies an energetic approach toward the social and material world and includes traits such as sociability, activity, assertiveness, and positive emotionality.” “*Agreeableness* contrasts a prosocial and communal orientation toward others with antagonism and includes traits such as altruism, tender-mindedness, trust, and modesty.” “*Conscientiousness* describes socially prescribed impulse control that facilitates task- and goal-directed behavior, such as thinking before acting, delaying gratification, following norms and rules, and planning, organising, and prioritising tasks.” “*Neuroticism* contrasts emotional stability and even-temperedness with negative emotionality, such as feeling anxious, nervous, sad, and tense.” “*Openness* to Experience (vs. closed-mindedness) describes the breadth, depth, originality, and complexity of an individual’s mental and experiential life.”	Stem: I see myself as someone who… is talkative. is outgoing, sociable.	7-point scale 1 (Strongly disagree) 2 (Disagree) 3 (Somewhat disagree) 4 (Neither agree nor disagree) 5 (Somewhat agree) 6 (Agree) 7 (Strongly agree)	Average of all items for each subscale.
Big-5 Personality trait– Agreeableness		Stem: I see myself as someone who… has a forgiving nature. is considerate and kind to almost everyone.		
Big-5 Personality trait– Conscientiousness		Stem: I see myself as someone who… does a thorough job. does things efficiently.		
Big-5 Personality trait– Neuroticism		Stem: I see myself as someone who… worries a lot. gets nervous easily.		
Big-5 Personality trait– Openness		Stem: I see myself as someone who… is original, comes up with new ideas. has an active imagination.		
**Level 2 (between-placements) Variables**				
Resilience	We utilised the 6-item Brief Resilience Scale (BRS) to assess self-perceptions regarding one’s ability to bounce back or recover from stress (B. W. Smith et al., 2008).	I tend to bounce back quickly after hard times. It does not take me long to recover from a stressful event.	5-point scale 1 (Strongly disagree) 2 (Disagree) 3 (Neutral) 4 (Agree) 5 (Strongly agree)	Average of all 6 items.
Motivation– Fear of Failure	We utilised the 5-item Performance Failure Appraisal Inventory (Short-Form), to measure the tendency to appraise threat or feel anxiety associated with the possibility of failing (Conroy et al., 2002).	When I am failing, I am afraid that I might not have enough talent. When I am failing, I worry about what others think about me.	5-point scale 1 (Do not believe at all) 2 (Believe 25% of the time) 3 (Believe 50% of the time) 4 (Believe 75% of the time) 5 (Believe 100% of the time)	Average of all 5 items.
Motivation– Intrinsic Goal Orientation	We adapted the 4-item Intrinsic Goal Orientation sub-scale from the Motivated Strategies of Learning Questionnaire (MSLQ) to suit a clinical context. Intrinsic orientations reflect goal pursuit for reasons such as challenge, curiosity or mastery (Pintrich et al., 1993).	The most satisfying thing for me in my clinical placements is trying to understand the content as thoroughly as possible. When I have the opportunity in my clinical placements, I choose experiences that I can learn from even if they don’t guarantee a good grade.	7-point scale 1 (Not at all true of me) 4 (Neutral) 7 (Very true of me)	Average of all 4 items.
Motivation– Extrinsic Goal Orientation	We adapted the 4-item Extrinsic Goal Orientation sub-scale from the Motivated Strategies of Learning Questionnaire (MSLQ) to suit a clinical context. Extrinsic orientations reflect goal pursuit for reasons such as grades, rewards, external evaluation or competition (Pintrich et al., 1993).	The most important thing for me right now is passing the next clinical placement. If I can, I want to get better grades in my clinical placement than most of the other students.	7-point scale 1 (Not at all true of me) 4 (Neutral) 7 (Very true of me)	Average of all 4 items.
Information Elaboration and Integration	We adapted the 6-item Elaboration sub-scale from the Motivated Strategies of Learning Questionnaire (MSLQ) to suit a clinical context. The information elaboration and integration scale captures strategies students may use to help integrate and connect new information with prior knowledge (Pintrich et al., 1993).	When I prepare for clinical placements, I pull together information from different sources, such as lectures, readings, and discussions. I try to understand the material in my physiotherapy degree by making connections between the readings and the concepts from the lectures.	7-point scale 1 (Not at all true of me) 4 (Neutral) 7 (Very true of me)	Average of all 6 items.
**Level 3 (within-placements) Variables**				
Academic Stressors	We created a study-specific measure based on a systematic review of stressors (Hurst et al., 2013) and past measurement of academic stressors amongst physiotherapy students (Tucker et al., 2006), to measure the number and frequency of stressors encountered. The preplacement survey (survey 1) included 18 items, whereas the surveys completed during placement weeks 1–5 (surveys 2–6) included 19 items.	Number /Frequency: Stem: This is a list of potential stressors that have been found to be highly applicable for university students. Thinking about your experiences over the past week, please indicate how often you experienced each of these potential stressors A disruptive or hostile learning environment. Adversity or major stressors related to the clinical placement (e.g., negative feedback, told you were failing).	8-point scale 0 (No days) 1 (On 1 Day) 2 (On 2 days) 3 (On 3 days) 4 (On 4 days) 5 (On 5 days) 6 (On 6 days) 7 (Every Day) Not Applicable	We calculated a summed score to reflect (1) total number of different stressors experienced and (2) their frequency of occurrence.
Adaptability	We utilised the 9-item Adaptability Scale to measure how an individual adjusts cognitively, affectively, and behaviourally when encountering uncertainty and novel circumstances (Martin et al., 2012).	To assist me in a new situation, I am able to change the way I do things if necessary. When uncertainty arises, I am able to minimise frustration or irritation so I can deal with it best.	7-point scale 1 (Strongly disagree) 2 (Disagree) 3 (Somewhat disagree) 4 (Neither agree nor disagree) 5 (Somewhat agree) 6 (Agree) 7 (Strongly agree)	Average of all 9 items.
Clinical Self-Efficacy	We created a 20-item study-specific scale, using Bandura’s (2006) Guide for constructing Self-Efficacy Scales and the Assessment of Physiotherapy Practice (APP) items (Dalton et al., 2009), to assess students’ belief in their ability to successfully demonstrate a range of entry-level physiotherapy clinical competencies.	Stem: On a scale of 0–100 rate your degree of confidence in being able to demonstrate each of these competencies at this point in your physiotherapy degree. Demonstrate collaborative practice (e.g., understand team processes and role of other health care professionals; advocate for client needs when dealing with other services). Conduct an appropriate client-centred interview (e.g., conducting a purposeful client interview to obtain relevant assessment information; respond/adapt effectively to client cues; identify clients’ goals and expectations).	11-point scale 0 (no confidence at all) 5 (moderate confidence) 10 (complete confidence)	Average of all 20 items.
Proactive Goal Regulation	We adapted the 12-item version of Bindl’s Proactive Goal Regulation Questionnaire (Bindl et al., 2012) to capture students’ perceptions of their efforts to bring about change based on anticipation of the future via envisioning, planning, enacting, and reflecting.	Stem: Thinking about how you have carried out your core university-related activities over the past week, how much effort have you spent… initiating better ways of studying, learning or preparing for clinical placements. seeking feedback from your clinical supervisors about the effects of your efforts to improve performance on clinical placements.	11-point scale 0 (0%) 1 (10%) 2 (20%) 3 (30%) 4 (40%) 5 (50%) 6 (60%) 7 (70%) 8 (80%) 9 (90%) 10 (100%)	Average of all items for each subscale.
Social Support - Availability	We created a bespoke 9-item tool in which students assessed the availability and amount of emotional, tangible, or informational support received or sought out from their family, friends, or romantic partner/spouse (where applicable).	Stem: Was this type of support available to you in the past week? Emotional support (e.g., expressions of love, empathy, trust, caring). Tangible (e.g., drive you to clinical placement, lend you money). Informational (e.g., advice, suggestions, feedback).	No Yes	We calculated a summed score to reflect the total number of support sources available to them.
Social Support - Seek		Stem: How often did you receive/seek out this type of support in the past week? Emotional support (e.g., expressions of love, empathy, trust, caring). Tangible (e.g., drive you to clinical placement, lend you money). Informational (e.g., advice, suggestions, feedback).	8-point scale 0 (No days) 1 (On 1 Day) 2 (On 2 days) 3 (On 3 days) 4 (On 4 days) 5 (On 5 days) 6 (On 6 days) 7 (Every Day)	We calculated a summed score to reflect the frequency with which support was received or sought out.

**Table 2:** Example of a single burst (5-week placement) with study variables and timeline

Measure/Tool	Pre-placement	Week 1 (Friday)	Week 2 (Friday)	Week 3 (Friday)	Week 4 (Friday)	Week 5 (Friday)
Demographic data	X (only first placement)					
Lifetime Adversity	X (only first placement)					
Resilience	X					
Big 5 Personality Index	X (only first placement)					
Academic Self Efficacy	X (only first placement)					
Clinical Self Efficacy	X	X	X	X	X	X
Academic Stressors	X	X	X	X	X	X
Adaptability	X	X	X	X	X	X
Motivational Orientations	X					
Proactive Goal Regulation	X	X	X	X	X	X
Persistence	X (only first placement)					
Perfectionism	X (only first placement)					
Information Elaboration and Integration	X					
Social Support	X	X	X	X	X	X
Clinical Performance						X

## Methods

### Ethics approval

This study was approved by Curtin University Human Research Ethics Committee (HRE2018-0509). All participants provided informed consent.

### Research context

At our institution, undergraduate Bachelor of Science (Physiotherapy) students complete approximately 1100 hours of clinical placements over the four-year degree. In the final year, students complete five 5-week, full-time placements, four of which are assessed by qualified clinical supervisors using the nationally-adopted Assessment of Physiotherapy Practice (APP) instrument (Dalton et al., 2009, 2011, 2012). All students complete APP-assessed placements in the following practice areas in random temporal order: cardiopulmonary, neurology, musculoskeletal, and other (miscellaneous category that includes clients across the lifespan from paediatrics to older adults). The fifth final year placement is a miscellaneous placement category, including project-based, international and inter-professional education (IPE) placements, which is typically evaluated using alternative assessment methods and instruments. Collectively, these placements provide opportunities for students to integrate theory and skills in real-world settings and, in doing so, prepare students for future practice.

### Design

**Fig. 1:**
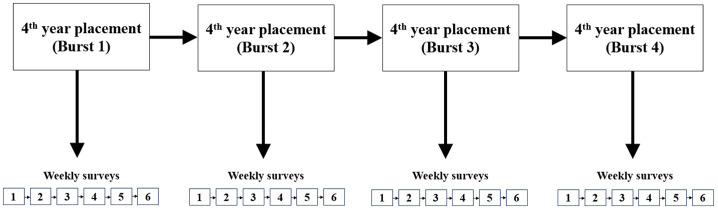
Visual depiction of measurement burst design study

We utilised a longitudinal measurement burst design, incorporating a hybrid of short-intensive and long-term assessments to examine intra-individual processes over time, as well as inter-individual differences related to these within-person dynamics (see Fig. 1) (Sliwinski, 2008). We prompted students to complete six weekly surveys for each 5-week placement across each APP-assessed placement in the final year of study. This design allows for enhanced precision (e.g., power to identify effects, assessment reliability) and scope (e.g., whether variables are temporally coupled within individuals) (Sliwinski, 2008).

### Participants

Our sample population included undergraduate physiotherapy students commencing their final year of study in 2019 and 2020 at Curtin University. Students provided informed consent electronically via a Qualtrics survey. Given our focus on within-person dynamics, for our analyses we retained consenting students who completed a minimum of three surveys per burst and at least two bursts in total. We provide an extended explanation of our sample size justification statement in supplementary file 3.

### Recruitment and data collection

 One of the authors (DG), not involved in teaching physiotherapy students, informed students about the study via face-to-face information sessions at lectures, with the support of 14 students who volunteered to ‘champion’ recruitment and engagement processes by disseminating information and messages via their student Facebook page. Prior to each clinical placement, consenting students received a link to the multi-section Qualtrics survey (Pre-placement survey/Survey 1) via their university student email address. At the end of every week (Friday) of each 5-week APP-assessed clinical placement, students received the weekly surveys and were requested to complete them prior to the start of the following week (Surveys 2–6). A research assistant, not involved in teaching, sent email and text message reminders to students who had not yet completed surveys after 48 hours, requesting completion by the Monday of the following week. Each pre-placement survey (Survey 1) took approximately 15–20 minutes to complete, whereas in-placement weekly surveys (Surveys 2–6) took approximately 5–10 minutes to complete. In total, students could complete up to 24 surveys. To minimise attrition and encourage sustained engagement, students who completed three or more surveys per burst received an AUD$20 gift voucher (David & Ware, 2014 ). Additionally, students who completed all 24 surveys, were entered into a prize draw to receive one of three AUD$100 gift vouchers (per cohort year).

### Primary outcome

The 20-item Assessment of Physiotherapy Practice (APP) was used as the primary outcome measure of student clinical performance across the 5-week placements (see supplementary material). This instrument is used to assess entry-level competencies aligned with the Australian and New Zealand physiotherapy standards (Physiotherapy Board of Australia & Physiotherapy Board of New Zealand, 2023). The APP consists of seven domains of clinical practice, including professional behaviour, communication, assessment, analysis and planning, intervention, evidence-based practice, and risk management. Clinical supervisors score each of the 20 items using a 5-point scale ranging from 0 (seldom meets criteria) to 4 (excellent performance) based on key performance descriptors. Full details of the development of the APP and initial validation are available elsewhere (Dalton et al., 2009, 2011, 2012). We operationalised clinical performance using the 2-factor representation (i.e. professional (items 1–4) and clinical (items 5–20) dimensions), based on recent psychometric evidence (Reubenson et al., 2020, 2025). We categorised the four APP-assessed placements into the following placement types: cardiopulmonary; musculoskeletal; neurological and other (miscellaneous category such as aged care, paediatrics).

### Determinants of clinical performance

We retrieved available student demographic data (e.g., date of birth, gender, course weighted average) from university databases. Self-reported psychosocial measures were obtained via each of the surveys completed within the measurement burst design. Regarding psychosocial determinants, we prioritised concepts that covered the multiple layers of psychological individuality (e.g., Big Five traits, self-efficacy, motivational orientations), self-regulatory factors (e.g., goal regulation, learning strategies), and social and contextual factors (e.g., stress exposure, social support). We selected operationalisations of these concepts for which there is sufficient reliability and validity evidence to support their use for our purposes. Full details of the target concepts and their operationalisations are provided in Table 1 and the supplementary material, with a visual representation of their implementation within the study design provided in Table 2.

### Statistical analysis

**Fig. 2:**
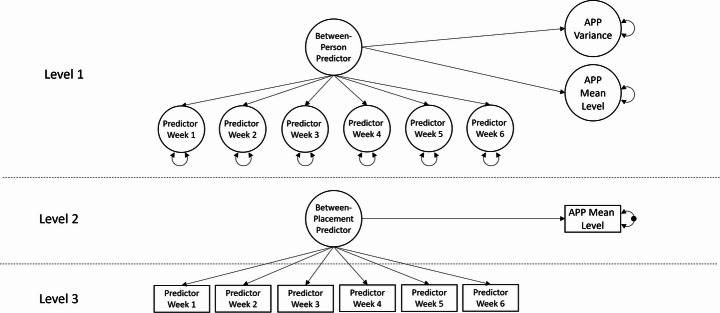
Latent variable decomposition of a within-placement predictor across between-placement and between-person levels. *Note:* Rectangles represent observed variables; unfilled circles represent latent variables. The black circle in the level 2 part of the model represents between-burst APP variance that is predicted at level 1. Additional level 1 observed variables are not depicted

We used multilevel structural equation modelling to account for nesting in our data, as responses collected for individual participants at repeated instances, both within- and between-placements, cannot be considered independent. We refer to data collected only at baseline as occurring *between-persons* (level 1); at the start or end of each placement as occurring *between-placements* (level 2); and each week during each placement as occurring *within-placements* (level 3). Our statistical model incorporated latent variable decomposition to partition variance across these three levels (see Fig. 2). We used a Bayesian estimator with default, non-informative priors to fit models in M*plus* 8.4 (Muthén & Muthén, 1998-2017) because of the computational benefits rather than for conceptual reasons (Asparouhov & Muthén, 2021).

We modelled observed predictor variables measured at the within-placement level (level 3) as indicators of a latent between-placement factor (level 2), which predicted placement-to-placement differences in clinical or professional APP scores. In the between-person (level 1) part of the model, we treated random intercepts from the within-placement level as latent indicators of a between-person factor, which predicted both person-to-person differences in mean levels (location component) of APP scores, as well as variability within-placements (scale component) modelled on the log scale (McNeish, 2021). We grand-mean centred level 1 variables (i.e., academic self-efficacy, perfectionism, lifetime adversity, persistence, and personality) and covariates to assess the influence of, and control for differences between students at baseline. As computational limitations prevented us from including all level 2 and 3 variables in a single model, we executed separate models for each psychosocial determinant. Models for between-placement predictors (i.e., those measured once per placement) varied slightly as there were no within-placement indicator variables. In these models, level 2 predictors were cluster mean centred and separated into between-placement and between-person components using latent variable decomposition. Similarly, we constructed separate models to test the effects of variables on clinical and professional dimensions of participants’ APP scores.

In accordance with best-practice guidelines (Pek & Flora, 2018), we report unstandardised estimates and 95% credible intervals for each model parameter. We consider parameters with credible intervals that exclude zero as compatible with a meaningful effect. For conciseness, we describe only results pertaining to predictors that were significantly associated with either mean levels or variability of APP scores. Readers unfamiliar with location-scale models can refer to supplementary file 3 for guidance on model interpretations.

## Results

### Flow of participants

**Fig. 3:**
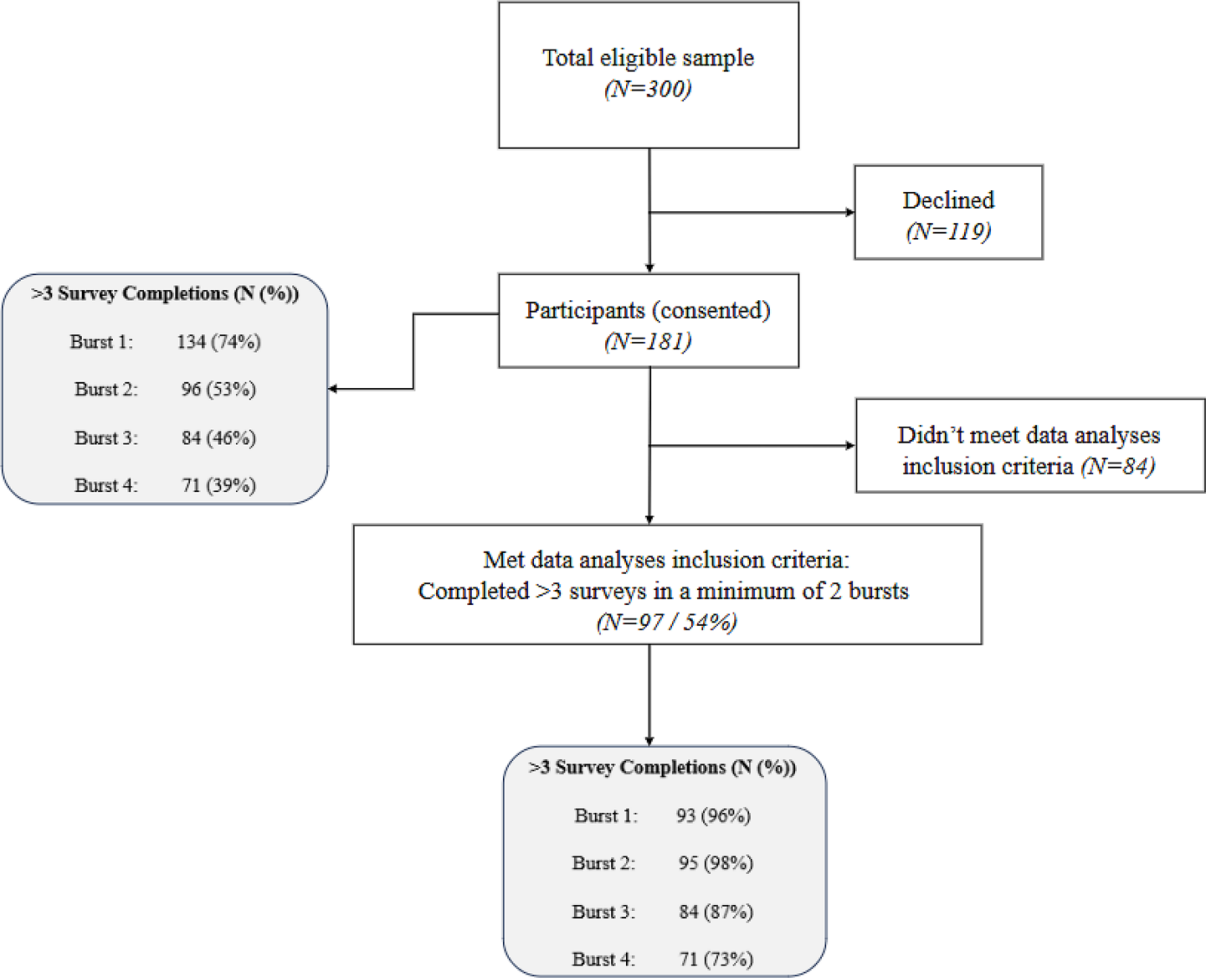
Flow of participants

In total, 300 students commenced final year clinical placements in 2019 (n = 141) and 2020 (n = 159) of whom 181 (60.3%) consented by completing survey 1 (pre-placement) prior to their first final year clinical placement. Of the 181 consenting students, 97 (53.6%) met the data analyses inclusion criteria by completing > 3 surveys in at least two bursts. Almost half (49.5%) of these students completed all 24 surveys. Retention across all four bursts ranged from 73–98%. Figure 3 and Table 3 provide a flow summary of participants and demographics, respectively.

**Table 3:** Participant demographics

Characteristic	Students (N = 97)
**Gender**^**a**^**, n (%)**	
Male	28 (28.9)
Female	69 (71.1)
**Honours, n (%)**	
Yes	16 (16.5)
No	81 (83.5)
**Age at 1st APP placement (y), mean (SD)**	24.3 (4.8)
**Citizenship, n (%)**	
Australian	73 (75.3)
International	24 (24.7)
**Main Language, n (%)**	
English	86 (88.7)
Non-English	11 (11.3)
**Course weighted average at end of course, mean (SD)**	73.6 (5)

^a^*Data often captured on entry to university programs; We acknowledge that students might identify in ways that differ from these two categories*

### Preliminary findings

Means, standard deviations, estimates of internal reliability, and bivariate correlations among study variables at the between-person and between-burst levels are reported in Table 4.

**Table 4:**
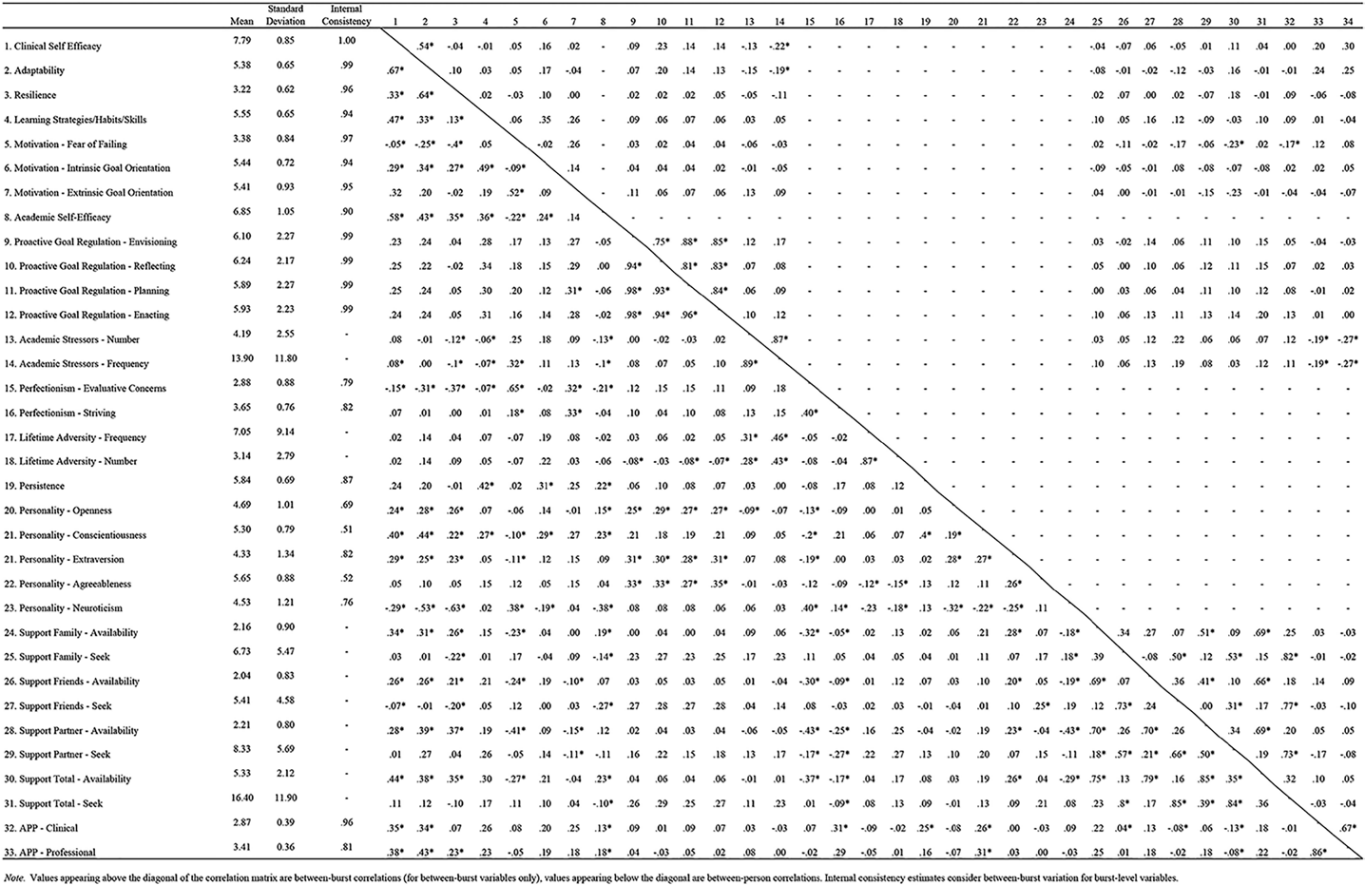
Descriptive statistics, internal consistency estimates, and correlations between study variables

**Table 5:** Effects of level 1 (between-person) covariates on clinical and professional dimensions of the APP

Effect	Clinical				Professional			
	Estimate	Posterior SD	CI Lower	CI Upper	Estimate	Posterior SD	CI Lower	CI Upper
**Location Fixed Effects**								
Intercept	2.921*	0.040	2.841	2.998	3.431*	0.045	3.339	3.513
Age	-0.027*	0.009	-0.044	-0.008	-0.194*	0.084	-0.345	-0.016
Course Weighted Average	0.011	0.009	-0.006	0.029	0.096	0.107	-0.106	0.312
Gender	-0.165	0.085	-0.330	0.005	-0.066	0.087	-0.236	0.106
Academic Self Efficacy	0.035	0.040	-0.044	0.115	0.012	0.100	-0.189	0.200
Perfectionism - Evaluative Concerns	0.013	0.048	-0.082	0.110	0.068	0.097	-0.125	0.248
Perfectionism - Striving	0.11*	0.054	0.003	0.216	0.135	0.099	-0.071	0.316
Lifetime Adversity - Frequency	-0.018	0.009	-0.036	0.000	-0.358*	0.144	-0.562	-0.005
Lifetime Adversity - Number	0.067*	0.027	0.012	0.121	0.344*	0.139	0.006	0.535
Persistence	-0.010	0.056	-0.120	0.101	0.060	0.090	-0.105	0.251
Personality - Openness	-0.025	0.039	-0.101	0.050	-0.014	0.086	-0.196	0.150
Personality - Conscientiousness	0.079	0.054	-0.023	0.184	0.135	0.093	-0.051	0.316
Personality - Extroversion	-0.018	0.029	-0.075	0.039	-0.026	0.091	-0.193	0.160
Personality - Agreeableness	0.012	0.044	-0.074	0.100	-0.038	0.087	-0.208	0.128
Personality - Neuroticism	-0.045	0.041	-0.125	0.034	-0.017	0.107	-0.226	0.190
**Location Random Intercept Covariance Structure**								
**Intercept**	0.038*	0.017	0.014	0.081	0.055*	0.019	0.030	0.106
**Scale Fixed Effects**								
Intercept	-1.43*	0.126	-1.705	-1.207	-1.492*	0.158	-1.793	-1.182
Age	0.040	0.023	-0.006	0.083	0.109	0.089	-0.075	0.278
Course Weighted Average	-0.007	0.027	-0.056	0.050	-0.056	0.099	-0.259	0.127
Gender	0.227	0.265	-0.246	0.795	0.063	0.084	-0.106	0.222
Academic Self Efficacy	-0.080	0.097	-0.270	0.117	-0.064	0.091	-0.253	0.103
Perfectionism - Evaluative Concerns	0.043	0.134	-0.245	0.298	-0.160	0.088	-0.327	0.010
Perfectionism - Striving	-0.205	0.162	-0.527	0.106	-0.091	0.088	-0.271	0.077
Lifetime Adversity - Frequency	0.039	0.021	-0.001	0.080	0.254	0.161	-0.105	0.507
Lifetime Adversity - Number	-0.120	0.069	-0.249	0.016	-0.233	0.157	-0.484	0.116
Persistence	-0.185	0.159	-0.510	0.119	-0.037	0.092	-0.223	0.139
Personality - Openness	0.161	0.098	-0.029	0.351	-0.007	0.081	-0.170	0.148
Personality - Conscientiousness	0.177	0.145	-0.107	0.455	-0.050	0.091	-0.229	0.130
Personality - Extroversion	0.015	0.080	-0.145	0.170	0.015	0.088	-0.156	0.188
Personality - Agreeableness	-0.149	0.117	-0.394	0.079	-0.053	0.084	-0.211	0.114
Personality - Neuroticism	0.159	0.127	-0.114	0.427	0.110	0.105	-0.112	0.298
**Scale Random Intercept Covariance Structure**								
Placement-Level Scale Variance	0.167*	0.112	0.053	0.467	1.025*	0.283	0.630	1.747
Corr(Intercept, Scale Variance)	-0.767*	0.245	-0.994	-0.117	-0.976*	0.041	-0.999	-0.852

SD = standard deviation; CI = credible interval, Corr = correlation,* 95% CI excludes zero

**Table 6:** Effects of level 3 (within-placement) variables on the clinical dimension of the APP

Effect	Adaptability				Academic Stressors (Number)				Academic Stressors (Frequency)				Clinical Self-Efficacy			
	Estimate	Posterior SD	CI Lower	CI Upper	Estimate	Posterior SD	CI Lower	CI Upper	Estimate	Posterior SD	CI Lower	CI Upper	Estimate	Posterior SD	CI Lower	CI Upper
**Location Fixed Effects**																
Intercept	2.904	0.043	2.817	2.985	2.917	0.041	2.835	2.997	2.919	0.040	2.837	2.997	2.909	0.044	2.822	2.993
Between-Person Effect	0.141*	0.057	0.030	0.252	0.017	0.041	-0.068	0.095	0.008	0.043	-0.076	0.096	-0.006	0.054	-0.107	0.103
Between-Placement Effect	0.279*	0.053	0.168	0.377	-0.189*	0.044	-0.274	-0.103	-0.181*	0.047	-0.271	-0.088	0.286*	0.048	0.196	0.383
**Location Random Intercept Covariance Structure**																
Intercept	0.034	0.016	0.010	0.073	0.031	0.017	0.008	0.073	0.031	0.017	0.010	0.074	0.035	0.017	0.009	0.074
Effect	0.070	0.036	0.021	0.160	0.048	0.024	0.017	0.108	0.056	0.025	0.022	0.119	0.059	0.027	0.024	0.130
Corr(Intercept, Effect)	-0.629	0.298	-0.937	0.208	0.296	0.334	-0.428	0.821	0.202	0.352	-0.522	0.823	-0.214	0.330	-0.789	0.460
**Scale Fixed Effects**																
Intercept	-1.970	0.294	-2.784	-1.595	-1.677	0.155	-1.973	-1.377	-1.709	0.148	-2.005	-1.429	-1.824	0.174	-2.220	-1.490
Between-Person Effect	-0.037	0.247	-0.588	0.396	0.065	0.125	-0.173	0.325	0.046	0.138	-0.196	0.342	0.058	0.154	-0.225	0.383
**Scale Random Intercept Covariance Structure**																
Placement-Level Scale Variance	0.329	0.339	0.038	1.307	0.242	0.142	0.053	0.590	0.254	0.141	0.050	0.584	0.187	0.159	0.023	0.609
Corr(Intercept, Scale Variance)	0.081	0.445	-0.736	0.883	-0.449	0.306	-0.887	0.261	-0.522	0.293	-0.916	0.179	-0.004	0.424	-0.785	0.767
Corr(Effect, Scale Variance)	-0.443	0.401	-0.906	0.610	0.299	0.368	-0.480	0.899	0.356	0.433	-0.652	0.895	-0.165	0.476	-0.853	0.851

SD = standard deviation; CI = credible interval, Corr = correlation; * 95% CI excludes zero

**Table 7:** Effects of level 3 (within-placement) variables on the professional dimension of the APP

	Adaptability				Academic Stressors (Number)				Academic Stressors (Frequency)				Clinical Self-Efficacy				Partner Support Seeking			
	Estimate	Posterior SD	CI Lower	CI Upper	Estimate	Posterior SD	CI Lower	CI Upper	Estimate	Posterior SD	CI Lower	CI Upper	Estimate	Posterior SD	CI Lower	CI Upper	Estimate	Posterior SD	CI Lower	CI Upper
**Location Fixed Effects**																				
Intercept	3.440	0.045	3.349	3.525	3.442	0.044	3.351	3.526	3.445	0.044	3.355	3.531	3.427	0.046	3.335	3.516	3.446	0.047	3.350	3.537
Between-Person Effect	0.105	0.057	-0.002	0.217	-0.007	0.043	-0.092	0.074	-0.007	0.047	-0.100	0.084	0.034	0.053	-0.070	0.138	-0.016	0.049	-0.118	0.076
Between-Placement Effect	0.249*	0.053	0.146	0.353	-0.138*	0.042	-0.219	-0.054	-0.142*	0.047	-0.238	-0.052	0.172*	0.047	0.079	0.263	-0.151*	0.066	-0.288	-0.025
**Location Random Intercept Covariance Structure**																				
Intercept	0.052	0.017	0.027	0.093	0.056	0.018	0.033	0.104	0.055	0.019	0.030	0.101	0.053	0.018	0.023	0.096	0.059	0.019	0.028	0.105
Effect	0.051	0.029	0.015	0.123	0.020	0.018	0.005	0.074	0.041	0.028	0.011	0.118	0.055	0.030	0.013	0.129	0.058	0.033	0.014	0.141
Corr(Intercept, Effect)	-0.637	0.225	-0.909	-0.074	0.444	0.305	-0.180	0.995	0.307	0.273	-0.257	0.794	-0.342	0.273	-0.819	0.240	0.461	0.309	-0.274	0.896
**Scale Fixed Effects**																				
Intercept	-1.892	0.220	-2.285	-1.424	-1.597	0.159	-1.945	-1.307	-1.707	0.175	-2.073	-1.377	-1.659	0.185	-2.059	-1.326	-1.790	0.239	-2.313	-1.391
Between-Person Effect	-0.443*	0.248	-0.959	-0.004	0.103	0.153	-0.192	0.421	0.066	0.172	-0.271	0.412	-0.076	0.211	-0.504	0.323	0.163	0.219	-0.248	0.606
**Scale Random Intercept Covariance Structure**																				
Placement-Level Scale Variance	0.830	0.391	0.202	1.781	0.862	0.310	0.354	1.586	0.908	0.355	0.359	1.714	0.846	0.376	0.355	1.803	1.015	0.459	0.430	2.180
Corr(Intercept, Scale Variance)	-0.881	0.153	-0.988	-0.423	-0.943	0.063	-0.994	-0.761	-0.945	0.074	-0.995	-0.728	-0.889	0.108	-0.988	-0.570	-0.907	0.101	-0.994	-0.622
Corr(Effect, Scale Variance)	0.313	0.305	-0.238	0.935	-0.350	0.337	-0.981	0.270	-0.211	0.292	-0.790	0.358	0.108	0.293	-0.449	0.738	-0.218	0.328	-0.791	0.431

SD = standard deviation; CI = credible interval, Corr = correlation; * 95% CI excludes zero

### Between-person determinants

Results for the level 1 covariates in the absence of level 2 or level 3 predictors are provided in Table 5. For simplicity, we exclude these level 1 effects in the reporting of results in the following sections for pragmatics given the large number of covariates, even though they are incorporated within all statistical models. Readers interested in the entire outputs for each analysis can access this information on our Open Science Framework (OSF) project page (https://osf.io/x8v3z).

The striving sub-dimension of perfectionism and the total number of adverse lifetime events positively predicted mean levels on the clinical dimension of performance, with individuals high in striving or with a higher number of total lifetime adverse events typically being assessed as performing better. Conversely, age negatively predicted mean levels of clinical performance, indicating that older participants performed more poorly. Regarding professional performance, age and frequency of adverse lifetime events were negatively associated with supervisor assessments of the professional dimension of the APP, whereas the number of adverse events were positively associated with the professional dimension of the APP.

### Between-placement determinants

There were no significant associations between level 2 variables (i.e., resilience and motivational factors) and mean levels of scores or between-placement variability in clinical or professional performance. Full details of these analyses are provided on the OSF project page.

### Within-placement determinants

Associations between level 3 variables that evidenced credible associations with clinical and professional dimensions of APP scores are provided in Tables 6 and 7, respectively. Adaptability had a positive association with clinical performance between-persons and between-placements, and professional performance between-placements. Students with higher average adaptability performed better in the clinical dimension than students with low average adaptability. Further, on placements where students reported higher week-to-week adaptability they performed better on both clinical and professional dimensions. Adaptability also negatively predicted intra-individual variability in professional performance, such that scores in the professional dimension tended to fluctuate less from placement-to-placement in students who reported higher levels of adaptability.

Experiencing a greater number of and more frequent academic stressors week-to-week while on a placement predicted lower performance for both clinical and professional dimensions for that placement compared to a student’s other placements. Conversely, students performed better on both clinical and professional dimensions in placements where they perceived or experienced greater clinical self-efficacy. Regarding the professional dimension only, students performed worse on placements where they reported seeking more support from their partner/spouse.

## Discussion

We investigated the salience of stable individual difference characteristics, and state-like psychosocial and self-regulatory factors as determinants of average levels and intra-individual variability in clinical performance between students and placements via a measurement-burst study design, which involved weekly surveys during each of four 5-week placements. Our findings supported inferences regarding the importance of both stable individual difference characteristics and self-regulatory factors. Key among these determinants is age, exposure to major lifetime adversities or academic stressors, perfectionistic strivings, beliefs regarding one’s efficacy to perform clinical skills well, support seeking, and self-perceived capacities to regulate one’s experiences with events characterised by novelty, change, or uncertainty.

### Conceptual and empirical contributions

Major lifetime and everyday events ranging from significant adversities to regular academic and life stressors and challenges are important contextual experiences for human functioning (Seery et al., 2010). Regarding cumulative lifetime adversities (e.g., personal and family illness and death, abuse and violence, discrimination, poverty) prior to study initiation, we found that the overall number of event categories experienced were positively associated with both the professional and clinical dimensions, whereas the frequency with which events were experienced were inversely associated with the professional dimension only. The magnitude of effect was strongest for the professional dimension when compared to the clinical dimension. Broadly, our findings align with the notion that moderate exposure to adversity, relative to no/low or high levels of adversity exposure, may offer a ‘steeling’ or strengthening effect that increases one’s resistance to future adversities (Höltge et al., 2018; Seery, 2011; Seery et al., 2010, 2013; Seery & Quinton, 2016). We use the analogy of exercise to illustrate how adversity can strengthen coping capacity: moderate stress promotes growth; excessive or insufficient stress impairs it; and recovery periods are essential for consolidating gains and restoring resources (Höltge et al., 2018). Essentially, lifetime adversity experiences shape and influence the quality and quantity of one’s acquired personal resource capabilities that students bring with them to life experiences, thus offering one vantage point into their propensity for regulating well when confronted with stressors that may translate into adaptive performance in clinical placement settings.

Regarding everyday stressors, we prioritised events at the interface between the academic environment and one’s life that are known to affect physiotherapy students (Gallasch et al., 2022; Tucker et al., 2006; Walsh et al., 2010). Self-reported during each 5-week placement, we found that students who were exposed to more stressor events and more frequently while on placement tended to perform worst on both professional and clinical dimensions. Exposure to multiple stressor events concurrently and/or sequentially within a certain time window (e.g., days, weeks)– often referred to as pileup– is common among adults (Schilling & Diehl, 2014; Serido et al., 2004). From the perspective of conservation of resources theory (Hobfoll et al., 2018), stressor pileup could deplete key psychological resources, such as self-efficacy and sense of autonomy/control, resulting in fewer reserves to manage both academic and clinical placement demands. The accumulation of stressors may increase perceived stress levels or overwhelm cognitive resources required for consolidating learning and clinical decision-making. In turn, perceived stress can impair attention, working-memory, self-regulatory capabilities, and impede students’ ability to recall key knowledge and apply new knowledge and skills effectively in complex, dynamic clinical environments (e.g. Gallasch et al., 2022; LeBlanc, 2009; Tam et al., 2025). Essentially, stressor event exposure during clinical placements characterises the degree to which students are required to self-regulate to function well and, in so doing, activate or deploy resources to regulate their functioning.

Individuals possess broad, stable traits characterised by reasonably consistent patterns of thinking, feeling, and behaving that shape how they engage within and across diverse social situations (McAdams, 2013). Among the dispositional factors assessed, perfectionistic strivings– setting and striving towards high personal standards of functioning– was the single credible determinant of the clinical dimension of performance only. Individuals with high perfectionistic strivings set ambitious personal goals and are often driven to achieve them through dedication, discipline, motivation, and perseverance (Damián Núñez et al., 2024; Madigan, 2019). In academic and sporting settings, these characteristics tend to result in improved adaptation, coping strategies, and competence (Damián Núñez et al., 2024; Endleman et al., 2022; Madigan, 2019; Ocampo et al., 2020). The association of perfectionist strivings with the clinical dimension of performance only is likely attributed to the more tangible nature of performance indicators within this dimension compared to the professional one, making it easier to set and strive towards relatable, objective goals. Nevertheless, our findings should be interpreted with caution, as high perfectionistic strivings, particularly in the presence of high perfectionistic evaluative concerns (e.g., attributing one’s self-worth to achievement, fear of making mistakes, self-doubt) may be associated with maladaptive psychological and wellbeing outcomes (Gaudreau et al., 2022; Smith et al., 2022). Further research is warranted to unpack the complexity that likely underlies the association between the multidimensional aspects of perfectionism and the clinical placement learning environment. This need is particularly pertinent given the rise in levels of perfectionism (Curran & Hill, 2019) and mental health disorders among the general public and student populations (Norton, 2023; Orygen, 2017; Wilkins et al., 2021).

Adaptability captures one’s capacity to regulate their thoughts, feelings, and behaviour effectively when confronted with events characterised by novelty, change, or uncertainty (Martin, 2012; Martin et al., 2012). Unlike resilience, which is operationalised via the Brief Resilience Scale as getting through and recovering from hard times, stressful events, and setbacks (Smith et al., 2008), adaptability references the mechanisms by which people seek to achieve positive outcomes. We found adaptability, and not resilience (cf. Calo et al., 2022), to be positively associated with the clinical dimension of physiotherapy practice performance across all four placements. Students who self-reported higher levels of adaptability performed better in both professional and clinical dimensions, within each 5-week clinical placement, and displayed lower variability of performance in the professional dimension of practice. Our findings are congruent with a substantial body of evidence supporting the adaptive nature of adaptability for student performance and well-being, both in terms of fostering positive outcomes (e.g., motivation, performance, life satisfaction) and protecting against negative outcomes (e.g., psychological distress, exhaustion) (Collie et al., 2017; Granziera et al., 2022; Holliman et al., 2019, 2021, 2022; Martin et al., 2024, 2013). Methodologically, we provide the first exemplar in which adaptability is assessed dynamically as a state-like concept, rather than statically as a trait-like concept, as has been done in most previous research. Considered alongside the credible inverse associations between stressor exposure and student performance, adaptability as a form of self-regulation capacity is a key determinant for students transitioning from structured and controlled campus-based learning environments into the dynamic and unpredictable, real-world dynamics of physiotherapy practice settings (Ferns et al., 2024; McGivern et al., 2024; Patton, 2018; Patton et al., 2013, 2018), where students need to balance work, life, and other study demands (Gallasch et al., 2022; Kurunsaari et al., 2022; Tucker et al., 2006; Walsh et al., 2010).

From a social cognitive theory perspective (Bandura, 2006), planning, initiating, and regulating action purposefully towards valued outcomes, and reflecting on our experiences to extract lessons learned relies heavily on one’s beliefs in their capacities to act and learn. Self-efficacy refers to one’s beliefs in how well they can perform specific tasks or actions to accomplish goals or objectives (Bandura, 2006). Efficacy beliefs can vary according to their level, generality, and strength (Bandura, 1986). We considered two forms of efficacy beliefs pertinent to the population and context of our investigation, namely academic and clinical self-efficacy beliefs. Broadly aligned with the specificity matching principle, where efficacy beliefs are expected to be most predictive of outcomes that are proximal in time and/or contextual elements (e.g., domain, task) (McWilliams et al., 2013; Valentine et al., 2004), we found that clinical self-efficacy beliefs but not academic self-efficacy beliefs were credible determinants of higher clinical performance. When students have higher clinical self-efficacy beliefs, they are more likely to expend effort and persevere in the face of clinical placement challenges and uncertainty, whereas individuals with lower clinical self-efficacy beliefs are more inclined to adopt avoidance behaviours, experience negative thoughts and feelings, and achieve poorer performance outcomes (Artino, 2012; Waddington, 2023). In our context, prior clinical performance experiences influence the formation of clinical self-efficacy beliefs, which in turn influence future clinical performance; over time, these reciprocal effects serve to calibrate one’s clinical self-efficacy beliefs (Bandura, 1997; Talsma et al., 2018). Taken together, our findings that adaptability and clinical self-efficacy beliefs are salient self-regulatory factors associated with positive clinical placement outcomes underscore the need to develop and bolster these personal resource elements prior to and throughout clinical placement sequences.

One’s perceptions of the level of assistance and interpersonal support available from key people in their social network is considered a conditional resource that varies in availability and utilisation, depending on temporal and contextual factors (Holliman et al., 2021). Although empirical findings are mixed, social support is generally associated with better academic performance (Richardson et al., 2012; Tindle et al., 2022) and diverse wellbeing outcomes (Biro et al., 2016; Holliman et al., 2021, 2022; Siedlecki et al., 2014). There is also limited reports in the nursing (Mergal et al., 2018) and physiotherapy (Gallasch et al., 2022; Paynter et al., 2023) literature suggesting positive effects (e.g., adaptive coping strategies) of social support mechanisms and clinical performance outcomes. Availability of social support resources are believed to act as a buffer against psychological distress in tertiary education settings (Crede & Niehorster, 2012; Gallasch et al., 2022; Holliman et al., 2021, 2022) and burnout in health work settings (Maresca et al., 2022). Of these different considerations of social support within our assessment package, the only credible effect was as an inverse association between partner/spouse seeking/receiving support and the professional dimension of practice. The non-experimental nature of our design precludes us from making causal inferences. For example, it could be that students who sought support from their partners found this assistance to be insufficient and therefore performed more poorly, or it was only when students were underperforming that they made use of the support available from their partners. Perhaps most important for future work on the salience of social support for student clinical performance, the type of support required works best when it ‘matches’ the type of stressors encountered (Mishra, 2020; Viswesvaran et al., 1999). We expect, for example, support from clinical supervisors rather than one’s family, friends, or spouses/partners is most likely to influence clinical performance, particularly when stressors occur in the work-integrated learning environment or ‘spill over’ to those settings (e.g., personal matters interfere with preparatory work before a challenging client consult). In developing interventions to support physiotherapy students prior to and during clinical placements, particular attention needs to be taken in matching the nature and type of support provided to the contextual demands at the time.

### Practical implications

Well-crafted, conceptually and empirically informed approaches to intervention in higher education settings can be effective and efficacious when they target specific educational problems and the mechanisms most likely to resolve them (Harackiewicz & Priniski, 2018). Stressor exposure and students’ ability to cope with change, novelty and uncertainty, and develop positive efficacy beliefs, are the most credible psychosocial factors likely to impact clinical performance, identified in our study. Throughout a physiotherapy degree, students will encounter multiple stressors, unique to the individual and their progression within the course. Therefore, curricular design providing structure with flexibility to support students to engage adaptively with stressors via meaningful and systematic reflective processes; and cultivate student agency to navigate challenging moments, and new and uncertain situations throughout the course, are potential considerations. As one example, the systematic self-reflection model of resilience strengthening (Crane et al., 2019b) encompasses five strategies targeting self-awareness of one’s cognitive, emotional, and behavioural experiences with stressors; event identification; (re)consideration of one’s initial appraisals; evaluation of the in/effectiveness of one’s response; and future focus to promote identification of new or refined coping strategies. Experimental evidence supports the utility of this framework among young adults (Crane et al., 2019a; Falon et al., 2021; Kho et al., 2023), including implementations led by training supervisors (Kho et al., 2023, 2024). Physiotherapy students could be introduced to the framework in early stages of the course and encouraged to follow the structured approach on a regular basis, with reinforcement of this meta-cognitive approach by clinical supervisors. Additionally, more targeted and guided coaching sessions could be embedded within the curriculum at known transition points and stressful times, such as prior to embarking on full-time clinical placements. Like other skills, repeated meta-cognitive engagement with stressor experiences is likely to enhance and refine students’ resilience capabilities including self-efficacy beliefs, adaptability and coping resources, and use of emotional self-regulatory repertoire (Bandura, 1997; Crane et al., 2019b; Delany et al., 2015; Martin et al., 2012, 2015).

### Strengths, limitations, and conclusions

Key strengths of this study include the integration of short-term and long-term repeated assessments of diverse psychosocial factors within an authentic, naturalistic workplace setting, and the separation of mean levels and within-person variability in clinical performance outcomes. The innovative measurement burst design permits insights into temporal dynamics in psychosocial and behavioural processes as students progressed through final year physiotherapy clinical placements, a key advantage over cross-sectional designs. Finally, our recruitment and retention strategies across a 2-year study period was effective in gaining a representative sample of final year physiotherapy students in our institution, lending credibility to study findings. Despite these strengths, our study is not without limitations. Our single-site observational study was exploratory in nature, limiting generalisability across other Australian and international physiotherapy education and health settings. Although we captured a diverse range of psychosocial and contextual factors, we examined each of the determinants independently and therefore the interactive effects between determinants warrants attention in future research. We also relied on purpose-built scales for self-efficacy, stressor exposure, and social support, as well as adapted others from ‘study’ broadly to the clinical performance context specifically. Doing so limits the extent to which our findings might generalise, noting generalisation was never an objective of our work. As with most observational studies, it is difficult to attribute causality and directionality to many of the observed associations; for example, the observed high striving levels might be an antecedent to clinical performance or an outcome of high clinical performance achievement (Endleman et al., 2022). We focussed solely on person-level factors; however, we acknowledge that clinical placement learning takes place in a complex socioecological framework (e.g., PPCT) and that a range of unobserved confounders related to students (e.g., living situation), supervisors (e.g., student-supervisor relationships, supervisor experience), the environment (e.g., placement organisational culture), the university (e.g., placement availability, quality of information and support provided to student and/or placement host), and legislative/accreditation requirements (e.g., mix of placement allocations) may influence performance in workplace settings (Ferns et al., 2024).

Learning and performance in complex workplace settings is known to be unpredictable and challenging. Students’ inherent and modifiable resources as well as environmental demands and encounters influence their behaviours, stress levels, and achievement within these surroundings. We provide an exemplar of a model to study this complexity that goes beyond the traditional methodologies used by researchers and universities and utilises a holistic approach that is more congruent with authentic clinical practice in which salient factors are influenced by multiple person-context interactions over time. Our findings highlight the importance of previous and concurrent stressors and the need to manage these in flexible ways. Educators should be encouraged to acknowledge the diversity of contemporary student populations and variability of practice settings, and design health science curricula to identify early students who may require additional personalised support whilst equipping all students with skills and strategies to effectively regulate their emotions, thoughts and behaviours when faced with novelty, change and uncertainty.

## Electronic supplementary material

Below is the link to the electronic supplementary material.

Supplementary Material 1: Supplementary Information File 1– APP Sample

Supplementary Material 2: Supplementary Information File 2– Survey questions

Supplementary Material 3: Supplementary Information File 3– Detailed statistical information

## Data Availability

Due to the sensitive nature of the data, namely student academic records, raw data is unavailable. Data are available on request from the Curtin University Human Research Ethics Committee (hrec@curtin.edu.au) for researchers who meet the criteria to gain access to confidential data.
