# Supplementary material for: Individual differences and self-regulatory factors are credible determinants of physiotherapy student performance on clinical placement: Insights from a measurement burst design study
Source: Adv Health Sci Educ Theory Pract. 2025 Aug 4;31(2):573–606. doi: 10.1007/s10459-025-10453-4 (PMC13046584; doi:10.1007/s10459-025-10453-4)
Supplement: Supplementary file 2 — Supplementary Material 2: Supplementary Information File 2– Survey questions [file 10459_2025_10453_MOESM2_ESM.docx]

| **Measure/Tool** | **Beginning of placement** | **Week 1 (Friday)** | **Week 2 (Friday)** | **Week 3 (Friday)** | **Week 4 (Friday)** | **Week 5 (Friday)** |
| --- | --- | --- | --- | --- | --- | --- |
| **Demographic data** | **X (only first placement)** |  |  |  |  |  |
| [**Lifetime Adversity**](#_Lifetime_Adversity) | **X (only first placement)** |  |  |  |  |  |
| [**Resilience**](#_Resilience_(Brief_Resilience) | **X** |  |  |  |  |  |
| [**Big 5 Personality Index**](#_Personality_(Short_15-item) | **X (only first placement)** |  |  |  |  |  |
| [**Academic Self Efficacy**](#_Academic_Self-efficacy) | **X (only first placement)** |  |  |  |  |  |
| [**Clinical Self Efficacy**](#_Clinical_Self-efficacy) | **X** | **X** | **X** | **X** | **X** | **X** |
| [**Academic Stressors**](#_Academic_Stressors) | **X** | **X** | **X** | **X** | **X** | **X** |
| [**Adaptability**](#_Adaptability_(Adaptability_Scale)) | **X** | **X** | **X** | **X** | **X** | **X** |
| [**Motivational Orientations**](#_Motivation_(Fear_of) | **X** |  |  |  |  |  |
| [**Proactive Goal Regulation**](#_Proactive_Goal_Regulation) | **X** | **X** | **X** | **X** | **X** | **X** |
| [**Persistence**](#_Persistence) | **X (only first placement)** |  |  |  |  |  |
| [**Perfectionism**](#_Perfectionism_(Frost_Multidimension) | **X (only first placement)** |  |  |  |  |  |
| [**Information**](#_Learning_Strategies,_Habits,) **Elaboration and Integration** | **X** |  |  |  |  |  |
| [**Social Support**](#_Social_Support_(Family;) | **X** | **X** | **X** | **X** | **X** | **X** |
| [**Clinical Performance**](#_Clinical_Competence) |  |  |  |  |  | **X** |

**Supplementary Information – Survey Questions**

| Academic Self-efficacy |  | |  | |  | |  |  | |  | | |  |  | |  | |  | |  | |
| --- | --- | --- | --- | --- | --- | --- | --- | --- | --- | --- | --- | --- | --- | --- | --- | --- | --- | --- | --- | --- | --- |
| **Instructions:** Below are a list of skills relevant to your studies.      On a scale of 0 - 100 rate your **degree of confidence** in... | No confidence at all | |  | |  | |  |  | | Moderate Confidence | | |  |  | |  | |  | | Complete Confidence | |
|  | 0 (0) | | 10 (1) | | 20 (2) | | 30 (3) | 40 (4) | | 50 (5) | | | 60 (6) | 70 (7) | | 80 (8) | | 90 (9) | | 100 (10) | |
| 1. Paying full attention to what is said or demonstrated by teaching staff during lectures/tutorials |  |  | |  | |  | | |  | |  |  | | |  | |  | |  | |  |
| 2. Breaking down complex information into simple, easy to remember concepts |  |  | |  | |  | | |  | |  |  | | |  | |  | |  | |  |
| 3. Asking questions to clarify your understanding when you are unsure of something in lectures/tutorials |  |  | |  | |  | | |  | |  |  | | |  | |  | |  | |  |
| 4. Extracting relevant information from learning resources (e.g., lectures, readings, demonstrations) |  |  | |  | |  | | |  | |  |  | | |  | |  | |  | |  |
| 5. Completing self-directed learning activities (e.g., practice questions, pre-readings) outside of formal teaching sessions (e.g., lectures, tutorials) |  |  | |  | |  | | |  | |  |  | | |  | |  | |  | |  |
| 6. Retaining large amounts of information |  |  | |  | |  | | |  | |  |  | | |  | |  | |  | |  |
| 7. Your ability to explain your understanding of concepts verbally |  |  | |  | |  | | |  | |  |  | | |  | |  | |  | |  |
| 8. Applying knowledge in new or different settings, situations, or subjects |  |  | |  | |  | | |  | |  |  | | |  | |  | |  | |  |
| 9. Your ability to convey your understanding of concepts accurately via written processes (e.g., assignments, exams) |  |  | |  | |  | | |  | |  |  | | |  | |  | |  | |  |
| 10. Critically analysing information or problems (e.g., consider all options and weigh up information in order to reach a fair-minded conclusion) |  |  | |  | |  | | |  | |  |  | | |  | |  | |  | |  |
| 11. Critiquing your own knowledge or skills with the view to identify learning needs |  |  | |  | |  | | |  | |  |  | | |  | |  | |  | |  |
| 12. Identifying solutions to a conceptual or practical problem (e.g., a case study) |  |  | |  | |  | | |  | |  |  | | |  | |  | |  | |  |
| 13. Acting effectively on your own self-reflection or feedback received from teaching staff or peers (e.g., doing things differently the next time around) |  |  | |  | |  | | |  | |  |  | | |  | |  | |  | |  |
| 14. Managing your emotional state (e.g., stress, anxiety) effectively during written examinations |  |  | |  | |  | | |  | |  |  | | |  | |  | |  | |  |
| 15. Effectively managing your workload commitments between study and other areas of your life (e.g., work, sports/hobbies/social, family) |  |  | |  | |  | | |  | |  |  | | |  | |  | |  | |  |
| 16. Managing your emotional state (e.g., stress, anxiety) effectively during practical examinations |  |  | |  | |  | | |  | |  |  | | |  | |  | |  | |  |

| Clinical Self-efficacy |  |  |  |  |  |  |  | |  |  |  |  |
| --- | --- | --- | --- | --- | --- | --- | --- | --- | --- | --- | --- | --- |
| **Instructions: Below are a list of competencies required to graduate as a physiotherapist in Australia. On a scale of 0 - 100 rate your degree of confidence in being able to demonstrate each of these competencies at this point in your physiotherapy degree.** | No confidence at all |  |  |  |  | Moderate Confidence | |  |  |  |  | Complete Confidence |
|  | 0 (0) | 10 (1) | 20 (2) | 30 (3) | 40 (4) | 50 (5) | | 60 (6) | 70 (7) | 80 (8) | 90 (9) | 100 (10) |
| **1. Demonstrate an understanding of client rights and consent** (e.g., obtain informed consent; understand and respect clients’ rights, privacy and dignity) |  |  |  |  |  |  |  | |  |  |  |  |
| **2. Demonstrate a commitment to learning** (e.g., take responsibility for own learning and seek out opportunities to meet learning needs; respond in a positive manner to questions, suggestions and/or constructive feedback) |  |  |  |  |  |  |  | |  |  |  |  |
| **3. Demonstrate ethical, legal and culturally responsive practice** (e.g., adhere to policies and procedures; maintain professional boundaries with clients) |  |  |  |  |  |  |  | |  |  |  |  |
| **4. Demonstrate collaborative practice** (e.g., understand team processes and role of other health care professionals; advocate for client needs when dealing with other services) |  |  |  |  |  |  |  | |  |  |  |  |
| **5. Communicate effectively with others** (e.g., use and adapt different verbal and non-verbal strategies with clients, family members, colleagues and/or other health professionals, taking into consideration aspects such as personal and language differences) |  |  |  |  |  |  |  | |  |  |  |  |
| **6. Demonstrate effective documentation** (e.g., accurately record clinical findings/information according to organisational and legislative requirements) |  |  |  |  |  |  |  | |  |  |  |  |
| **7. Conduct an appropriate client-centred interview** (e.g., conducting a purposeful client interview to obtain relevant assessment information; respond/adapt effectively to client cues; identify clients’ goals and expectations) |  |  |  |  |  |  |  | |  |  |  |  |
| **8. Measure relevant health indicators and outcomes** (e.g., select appropriate variables to be measured from the World Health Organisation (WHO) International Classification of Functioning, Disability and Health (ICF) domains of activity limitation and participation restriction; able to monitor treatment response and outcomes) |  |  |  |  |  |  |  | |  |  |  |  |
| **9. Perform an appropriate physical/objective assessment** (e.g., complete a systematic, purposeful and safe physical/objective examination of a client; demonstrate sensitive and appropriate handling during the assessment process) |  |  |  |  |  |  |  | |  |  |  |  |
| **10. Interpret assessment findings appropriately** (e.g., describe the implications of test results; relate signs and symptoms to pathology; prioritise important assessment findings) |  |  |  |  |  |  |  | |  |  |  |  |
| **11. Prioritise a client’s main problems** (e.g., generate a list of problems for the assessment; consider client’s values, priorities and needs) |  |  |  |  |  |  |  | |  |  |  |  |
| **12. Set realistic short-term and long-term goals in collaboration with a client** (e.g., in negotiation with client, formulate short and long term goals that are specific, measurable, achievable and relevant) |  |  |  |  |  |  |  | |  |  |  |  |
| **13. Select appropriate intervention/management plans that address the main client problems and goals** (e.g., options for physiotherapy input are identified and justified, based on client needs, on best evidence and available resources; demonstrates understanding of contraindications and precautions in selection of intervention/management strategies) |  |  |  |  |  |  |  | |  |  |  |  |
| **14. Perform interventions appropriately** (e.g., demonstrate appropriate handling skills; perform interventions in a safe manner) |  |  |  |  |  |  |  | |  |  |  |  |
| **15. Be an effective educator/health promoter** (e.g., provide relevant information with consideration of clients’ needs; discuss expectations of physiotherapy intervention/management and its intended outcomes; demonstrates skill in client education by modifying approach to suit the client age group and level of understanding) |  |  |  |  |  |  |  | |  |  |  |  |
| **16. Monitor the effects of interventions** (e.g., incorporate relevant evaluation procedures/outcome measures within the physiotherapy plan; make modifications to intervention based on evaluation) |  |  |  |  |  |  |  | |  |  |  |  |
| **17. Progress/regress treatment/management based on ongoing evaluations** (e.g., safe and sensible treatment modifications are made in consultation with the client based on best available evidence; treatment is discontinued in the absence of measurable benefit) |  |  |  |  |  |  |  | |  |  |  |  |
| **18. Undertake discharge planning** (e.g., planning for discharging a client from physiotherapy care; address client and carer needs for ongoing care through the coordination of appropriate services; describe strategies that may be useful for maintaining and improving health status following discharge) |  |  |  |  |  |  |  | |  |  |  |  |
| **19. Apply evidence-based practice in client-centred care** (e.g., consider the research evidence, client preferences, clinical expertise and available resources when making decisions about client management; locate and apply relevant current evidence, such as clinical practice guidelines, into practice) |  |  |  |  |  |  |  | |  |  |  |  |
| **20. Perform duties in a safe manner and minimise any risk associated with assessment and interventions** (e.g., monitor client safety during assessment and treatment; describe relevant contraindications and precautions associated with assessment and treatment) |  |  |  |  |  |  |  | |  |  |  |  |

| Academic Stressors |  |  | | | | |
| --- | --- | --- | --- | --- | --- | --- |
| Instructions: This is a list of potential stressors that have been found to be highly applicable for university students. Thinking about your experiences over the past week, please indicate (i) how often you experienced each of these potential stressors and (ii) the degree to which you interpreted each as being a challenge (i.e., can potentially contribute to your personal development) or hindrance (i.e., interferes with your academic goals and likelihood of success) for your performance.    Unless you select 'No days' or 'Not Applicable' in the first column, please complete both columns for each item. | **How often did this potential stressor occur in the past week?**  ▼ No days (1 ... Not Applicable (-99) | **How did you interpret this potential stressor?** | | | | |
|  |  | 100% hindrance (interferes with academic development/goals) (1) | 75% hindrance (2) | 50% hindrance / 50% challenge (3) | 75% challenge (4) | 100% challenge (potential to benefit academic development/goals) (5) |
| 1) Issues with your family | ▼ No days (1 ... Not Applicable (-99) |  |  |  |  |  |
| 2) Issues with your partner (e.g., boy/girlfriend/spouse) | ▼ No days (1 ... Not Applicable (-99) |  |  |  |  |  |
| 3) Issues with your friends | ▼ No days (1 ... Not Applicable (-99) |  |  |  |  |  |
| 4) Administrative issues at university (e.g., timetabling) | ▼ No days (1 ... Not Applicable (-99) |  |  |  |  |  |
| 5) Lack of time | ▼ No days (1 ... Not Applicable (-99) |  |  |  |  |  |
| 6) Financial issues (e.g., university fees; living expenses) | ▼ No days (1 ... Not Applicable (-99) |  |  |  |  |  |
| 7) Inadequate academic support from clinical placement supervisor/s | ▼ No days (1 ... Not Applicable (-99) |  |  |  |  |  |
| 8) Inadequate academic support from university staff (e.g., clinical coordinator) | ▼ No days (1 ... Not Applicable (-99) |  |  |  |  |  |
| 9) Issues with accommodation or living arrangements | ▼ No days (1 ... Not Applicable (-99) |  |  |  |  |  |
| 10) Self-expectations | ▼ No days (1 ... Not Applicable (-99) |  |  |  |  |  |
| 11) Others’ expectations | ▼ No days (1 ... Not Applicable (-99) |  |  |  |  |  |
| 12) Academic or coursework demands | ▼ No days (1 ... Not Applicable (-99) |  |  |  |  |  |
| 13) A disruptive or hostile learning environment | ▼ No days (1 ... Not Applicable (-99) |  |  |  |  |  |
| 14) Being in an unfamiliar learning environment | ▼ No days (1 ... Not Applicable (-99) |  |  |  |  |  |
| 15) Future career aspirations | ▼ No days (1 ... Not Applicable (-99) |  |  |  |  |  |
| 16) Extracurricular activities (e.g., sport, paid work) | ▼ No days (1 ... Not Applicable (-99) |  |  |  |  |  |
| 17) Health concerns (physical or psychological) | ▼ No days (1 ... Not Applicable (-99) |  |  |  |  |  |
| 18) Diversity-related concerns (e.g., your religious views, sexual orientation, race) | ▼ No days (1 ... Not Applicable (-99) |  |  |  |  |  |

| 19) Adversity or major stressors related to the clinical placement (e.g., negative feedback, told you were failing) | ▼ No days (1 ... Not Applicable (-99) |  |  |  |  |  |
| --- | --- | --- | --- | --- | --- | --- |

| Adaptability (Adaptability Scale) |  |  |  |  |  |  |  |
| --- | --- | --- | --- | --- | --- | --- | --- |
| For each of the statements below, please indicate which best applies to you. | Strongly disagree (1) | Disagree (2) | Somewhat disagree (3) | Neither agree nor disagree (4) | Somewhat agree (5) | Agree (6) | Strongly agree (7) |
| 1. I am able to think through a number of possible options to assist me in a new situation |  |  |  |  |  |  |  |
| 2. I am able to revise the way I think about a new situation to help me through it |  |  |  |  |  |  |  |
| 3. I am able to adjust my thinking or expectations to assist me in a new situation if necessary |  |  |  |  |  |  |  |
| 4. I am able to seek out new information, helpful people, or useful resources to effectively deal with new situations |  |  |  |  |  |  |  |
| 5. In uncertain situations, I am able to develop new ways of going about things (e.g., a different way of asking questions or finding information) to help me through |  |  |  |  |  |  |  |
| 6. To assist me in a new situation, I am able to change the way I do things if necessary |  |  |  |  |  |  |  |
| 7. I am able to reduce negative emotions (e.g., fear) to help me deal with uncertain situations |  |  |  |  |  |  |  |
| 8. When uncertainty arises, I am able to minimise frustration or irritation so I can deal with it best |  |  |  |  |  |  |  |
| 9. To help me through new situations, I am able to draw on positive feelings and emotions (e.g., enjoyment, satisfaction) |  |  |  |  |  |  |  |

| Resilience (Brief Resilience Scale) |  |  |  |  |  |
| --- | --- | --- | --- | --- | --- |
| **Instructions:** Use the following scale to indicate how much you disagree or agree with each of the statements | Strongly disagree (1) | Disagree (2) | Neutral (3) | Agree (4) | Strongly agree (5) |
| 1. I tend to bounce back quickly after hard times |  |  |  |  |  |
| 2. I have a hard time making it through stressful events |  |  |  |  |  |
| 3. It does not take me long to recover from a stressful event |  |  |  |  |  |
| 4. It is hard for me to snap back when something bad |  |  |  |  |  |
| 5. I usually come through difficult times with little trouble |  |  |  |  |  |
| 6. I tend to take a long time to get over set-backs in my life |  |  |  |  |  |

| Perfectionism (Frost Multidimensional Perfectionism Scale–Brief) |  |  |  |  |  |
| --- | --- | --- | --- | --- | --- |
| For the following statements, please indicate to what extent you agree or disagree with the statement. | Strongly disagree (1) | Disagree (2) | Neither agree nor disagree (3) | Agree (4) | Strongly agree (5) |
| 1. If I fail at work/university, I am a failure as a person |  |  |  |  |  |
| 2. I set higher goals for myself than most people |  |  |  |  |  |
| 3. If someone does a task at work/university better than me, then I feel like I failed at the whole task |  |  |  |  |  |
| 4. I have extremely high goals |  |  |  |  |  |
| 5. Other people seem to accept lower standards from themselves than I do |  |  |  |  |  |
| 6. If I do not do well all the time, people will not respect me |  |  |  |  |  |
| 7. I expect higher performance in my daily tasks than most people |  |  |  |  |  |
| 8. The fewer mistakes I make, the more people will like me |  |  |  |  |  |

| Information Elaboration and Integration |  |  |  |  |  |  |  |
| --- | --- | --- | --- | --- | --- | --- | --- |
| The following questions ask about your learning strategies and study skills.  For each statement indicate how much it relates to you. | 1 (1) | 2 (2) | 3 (3) | 4 (4) | 5 (5) | 6 (6) | 7 (7) |
| 1. When I prepare for clinical placements, I pull together information from different sources, such as lectures, readings, and discussions |  |  |  |  |  |  |  |
| 2. I try to relate ideas encountered on a clinical placement to those in other units in the course whenever possible |  |  |  |  |  |  |  |
| 3. When reading material in my physiotherapy degree, I try to relate the material to what I already know |  |  |  |  |  |  |  |
| 4. When I study for my physiotherapy degree, I write brief summaries of the main ideas from the readings and the concepts from the lectures |  |  |  |  |  |  |  |
| 5. I try to understand the material in my physiotherapy degree by making connections between the readings and the concepts from the lectures |  |  |  |  |  |  |  |
| 6. I try to apply ideas from course readings in other activities such as clinical placements |  |  |  |  |  |  |  |

| Lifetime Adversity |  |  | |
| --- | --- | --- | --- |
| **Instructions:** We'd like to ask you about some events that may have happened to you **during your lifetime**. Please indicate if you have ever in your life experienced any of the following events by selecting "No" or "Yes"? For those events you have experienced, please indicate how many times you have experienced the event? If you cannot recall the specific number of times an event has occurred, please provide an estimation. For those events that you haven't experienced, please select "Not Applicable" from the drop-down list in the "Number of times experienced" column. | Number of times experienced | Have you experienced this event? | |
|  |  | No (0) | Yes (1) |
| Major illness (physical or psychological) | ▼ 1 (1 ... Not Applicable (-99) |  |  |
| Life threatening accident | ▼ 1 (1 ... Not Applicable (-99) |  |  |
| Fire, flood or other natural disaster | ▼ 1 (1 ... Not Applicable (-99) |  |  |
| Witnessed someone badly injured or killed | ▼ 1 (1 ... Not Applicable (-99) |  |  |
| Parents divorced | ▼ 1 (1 ... Not Applicable (-99) |  |  |
| Sexual abuse | ▼ 1 (1 ... Not Applicable (-99) |  |  |
| Serious physical attack or assault | ▼ 1 (1 ... Not Applicable (-99) |  |  |
| Threatened/harassed without a weapon | ▼ 1 (1 ... Not Applicable (-99) |  |  |
| Threatened/harassed with a weapon/held | ▼ 1 (1 ... Not Applicable (-99) |  |  |
| Tortured or victim of terrorists | ▼ 1 (1 ... Not Applicable (-99) |  |  |
| Domestic violence | ▼ 1 (1 ... Not Applicable (-99) |  |  |
| Witnessed domestic violence | ▼ 1 (1 ... Not Applicable (-99) |  |  |
| Death of a loved one (e.g., parent, sibling) | ▼ 1 (1 ... Not Applicable (-99) |  |  |
| Serious illness or accident of a loved one (e.g., parent, sibling) | ▼ 1 (1 ... Not Applicable (-99) |  |  |
| Discrimination because of your ethnicity, religious background, or sexual orientation | ▼ 1 (1 ... Not Applicable (-99) |  |  |
| Drug or alcohol addiction | ▼ 1 (1 ... Not Applicable (-99) |  |  |
| Serious financial difficulties (e.g., no money for food or housing) | ▼ 1 (1 ... Not Applicable (-99) |  |  |
| Witnessed someone suicide or attempt suicide | ▼ 1 (1 ... Not Applicable (-99) |  |  |
| Child abuse – physical | ▼ 1 (1 ... Not Applicable (-99) |  |  |

Have you experienced any other stressful event (not listed above)?

- Yes (1)
- No (0)

Please specify what other stressful event/s you have experienced in the 'Other stressful event' box below. For each entry please also estimate how many times you have experienced the event? If you cannot recall the specific number of times an event occurred, please provide an estimation.

|  | Number of times experienced |
| --- | --- |
|  |  |
| Other stressful event 1 | ▼ 1 (1 ... 6+ (6) |
| Other stressful event 2 | ▼ 1 (1 ... 6+ (6) |
| Other stressful event 3 | ▼ 1 (1 ... 6+ (6) |

# Motivational Orientations (Fear of Failure, Intrinsic Goal Orientation, Extrinsic Goal Orientation)

| Motivation: Fear of Failure |  |  |  |  |  |
| --- | --- | --- | --- | --- | --- |
| For each of these statements indicate to what extent you agree with the statement on the scale provided. | Do not believe at all (1) | Believe 25% of the time (2) | Believe 50% of the time (3) | Believe 75% of the time (4) | Believe 100% of the time (5) |
| 1. When I am failing, I worry about what others think about me |  |  |  |  |  |
| 2. When I am failing, I am afraid that I might not have enough talent |  |  |  |  |  |
| 3. When I am failing, it upsets my “plan” for the future |  |  |  |  |  |
| 4. When I am not succeeding, people are less interested in me |  |  |  |  |  |
| 5. When I am failing, important others are disappointed |  |  |  |  |  |

| Motivation: Intrinsic Goal Orientation |  |  | | | | |  |
| --- | --- | --- | --- | --- | --- | --- | --- |
| For each statement indicate how much it relates to you. |  | The following questions ask about your motivation towards your studies. | | | | |  |
|  | 1 (1)  Not at all true of me | 2 (2) | 3 (3) | 4 (4)  Neutral | 5 (5) | 6 (6) | 7 (7)  Very true of me |
| 1. During clinical placements, I prefer experiences that really challenge me so I can learn new things |  |  |  |  |  |  |  |
| 2. During clinical placements, I prefer experiences that spark my curiosity, even if they are difficult to deal with |  |  |  |  |  |  |  |
| 3. The most satisfying thing for me in my clinical placements is trying to understand the content as thoroughly as possible |  |  |  |  |  |  |  |
| 4. When I have the opportunity in my clinical placements, I choose experiences that I can learn from even if they don't guarantee a good grade |  |  |  |  |  |  |  |

| Motivation: Extrinsic Goal Orientation |  |  | | | | |  |
| --- | --- | --- | --- | --- | --- | --- | --- |
| For each statement indicate how much it relates to you | Not at all true of me | The following questions ask about your motivation towards your studies.  Neutral | | | | | Very true of me |
|  | 1 (1) | 2 (2) | 3 (3) | 4 (4) | 5 (5) | 6 (6) | 7 (7) |
| 1. Getting a good grade in my clinical placement is the most satisfying thing for me right now |  |  |  |  |  |  |  |
| 2. The most important thing for me right now is passing the next clinical placement |  |  |  |  |  |  |  |
| 3. If I can, I want to get better grades in my clinical placement than most of the other students |  |  |  |  |  |  |  |
| 4. I want to do well in my clinical placement because it is important to show my ability to my family, friends, employer, or others |  |  |  |  |  |  |  |

| Persistence |  |  |  |  |  |  |  |
| --- | --- | --- | --- | --- | --- | --- | --- |
| For the following statements, please indicate to what extent you agree or disagree with the statement. | Strongly disagree (1) | Disagree (2) | Somewhat disagree (3) | Neither agree nor disagree (4) | Somewhat agree (5) | Agree (6) | Strongly agree (7) |
| 1. If I can’t understand my university work at first, I keep going over it until I do |  |  |  |  |  |  |  |
| 2. If an assignment is difficult, I keep working at it to figure it out |  |  |  |  |  |  |  |
| 3. When I’m taught something that doesn’t make sense, I spend time to try and understand it |  |  |  |  |  |  |  |
| 4. I’ll keep working at difficult university work until I think I’ve worked it out |  |  |  |  |  |  |  |

| Proactive Goal Regulation | | Instructions: There are many ways by which students attempt to reach their goals and perform to the best of their ability. Below are statements that describe some of these processes. Using the scale below, please indicate the extent to which you believe each statement is an indication of your own motives. | | | | | | | | | | | | | | | | |
| --- | --- | --- | --- | --- | --- | --- | --- | --- | --- | --- | --- | --- | --- | --- | --- | --- | --- | --- |
| *Thinking about how you have carried out your core university-related activities over the past week, how much effort have you spent...* | | 0% (0) | | 10% (1) | 20% (2) | | 30% (3) | 40% (4) | | 50% (5) | 60% (6) | | 70% (7) | 80% (8) | | 90% (9) | | 100% (10) |
| making changes to the ways you study, learn or prepare for clinical placements | |  | |  |  | |  |  | |  |  | |  |  | |  | |  |
| initiating better ways of studying, learning or preparing for clinical placements | |  | |  |  | |  |  | |  |  | |  |  | |  | |  |
| coming up with new ideas to improve the ways by which you study, learn or prepare for clinical placements | |  | |  |  | |  |  | |  |  | |  |  | |  | |  |
| thinking about ways to improve the ways by which you study key material outside of clinical placements | |  | |  |  | |  |  | |  |  | |  |  | |  | |  |
| going through different scenarios in your head about how best to bring about improvements in the ways by which you perform on clinical placements | |  | |  |  | |  |  | |  |  | |  |  | |  | |  |
| monitoring or assessing the effects of your efforts to improve the ways you study, learn or prepare for clinical placements | |  | |  |  | |  |  | |  |  | |  |  | |  | |  |
| thinking about ways to improve the ways by which you learn on clinical placements | |  | |  |  | |  |  | |  |  | |  |  | |  | |  |
| getting yourself into the ‘ideal zone’ (e.g., mood) before trying to make a change as to how you study, learn or prepare for clinical placements | |  | |  |  | |  |  | |  |  | |  |  | |  | |  |
| seeking feedback from your clinical supervisors about the effects of your efforts to improve performance on clinical placements | |  | |  |  | |  |  | |  |  | |  |  | |  | |  |
| thinking about ways to improve the way you prepare for clinical placements | |  | |  |  | |  |  | |  |  | |  |  | |  | |  |
| considering the pros and cons of different strategies to improving your study, learning or preparation for clinical placements | |  | |  |  | |  |  | |  |  | |  |  | |  | |  |
| learning from your efforts to improve the ways you study, learn or prepare for clinical placements (e.g., identifying strategies that worked and didn’t work) | |  | |  |  | |  |  | |  |  | |  |  | |  | |  |
| Personality (Short 15-item Big Five Inventory: BFI-S) |  | |  | | |  | | |  | | |  | | |  | |  | |
| Below are a number of statements, each of which starts with "I see myself as someone who...".  For each statement, indicate how much you agree with this. | Strongly disagree (1) | | Disagree (2) | | | Somewhat disagree (3) | | | Neither agree nor disagree (4) | | | Somewhat agree (5) | | | Agree (6) | | Strongly agree (7) | |
| worries a lot |  | |  | | |  | | |  | | |  | | |  | |  | |
| gets nervous easily |  | |  | | |  | | |  | | |  | | |  | |  | |
| remains calm in tense situations |  | |  | | |  | | |  | | |  | | |  | |  | |
| is talkative |  | |  | | |  | | |  | | |  | | |  | |  | |
| is outgoing, sociable |  | |  | | |  | | |  | | |  | | |  | |  | |
| is reserved |  | |  | | |  | | |  | | |  | | |  | |  | |
| is original, comes up with new ideas |  | |  | | |  | | |  | | |  | | |  | |  | |
| values artistic, aesthetic experiences |  | |  | | |  | | |  | | |  | | |  | |  | |
| has an active imagination |  | |  | | |  | | |  | | |  | | |  | |  | |
| is sometimes rude to others |  | |  | | |  | | |  | | |  | | |  | |  | |
| has a forgiving nature |  | |  | | |  | | |  | | |  | | |  | |  | |
| is considerate and kind to almost everyone |  | |  | | |  | | |  | | |  | | |  | |  | |
| does a thorough job |  | |  | | |  | | |  | | |  | | |  | |  | |
| tends to be lazy |  | |  | | |  | | |  | | |  | | |  | |  | |
| does things efficiently |  | |  | | |  | | |  | | |  | | |  | |  | |

# Social Support (Family; Friends; Spouse/Partner)

| Social Support (Family) | **Instructions:** Thinking about your experiences **over the past week**, please indicate (i) whether you felt this type of support was available to you and (ii) how often did you receive/seek out this type of support? | | | | | | | |  | |
| --- | --- | --- | --- | --- | --- | --- | --- | --- | --- | --- |
| The following 3 statements relate to support from your **family**  Please complete both columns for each item. | How often did you receive/seek out this type of support in the past week? | | | | | | | | Was this type of support available to you in the past week? | |
|  | No days (0) | On 1 day (1) | On 2 days (2) | On 3 days (3) | On 4 days (4) | On 5 days (5) | On 6 days (6) | Every day (7) | Yes (1) | No (0) |
| Emotional support (e.g., expressions of love, empathy, trust, caring) |  |  |  |  |  |  |  |  |  |  |
| Tangible (e.g., drive you to clinical placement, lend you money) |  |  |  |  |  |  |  |  |  |  |
| Informational (e.g., advice, suggestions, feedback) |  |  |  |  |  |  |  |  |  |  |

| Social Support (Friends) | **Instructions:** Thinking about your experiences **over the past week**, please indicate (i) whether you felt this type of support was available to you and (ii) how often did you receive/seek out this type of support? | | | | | | | |  | |
| --- | --- | --- | --- | --- | --- | --- | --- | --- | --- | --- |
| The following 3 statements relate to support from your **friends.** Please complete both columns for each item. | How often did you receive/seek out this type of support in the past week? | | | | | | | | Was this type of support available to you in the past week? | |
|  | No days (0) | On 1 day (1) | On 2 days (2) | On 3 days (3) | On 4 days (4) | On 5 days (5) | On 6 days (6) | Every day (7) | Yes (1) | No (0) |
| Emotional support (e.g., expressions of love, empathy, trust, caring) |  |  |  |  |  |  |  |  |  |  |
| Tangible (e.g., drive you to clinical placement, lend you money) |  |  |  |  |  |  |  |  |  |  |
| Informational (e.g., advice, suggestions, feedback) |  |  |  |  |  |  |  |  |  |  |

Q4 Do you have a **partner/spouse**?

- Yes (1)
- No (0)

| Social Support (Partner/Spouse) | **Instructions:** Thinking about your experiences **over the past week**, please indicate (i) whether you felt this type of support was available to you and (ii) how often did you receive/seek out this type of support? | | | | | | | |  | |
| --- | --- | --- | --- | --- | --- | --- | --- | --- | --- | --- |
| The following 3 statements relate to support from your **partner/spouse** (if applicable) Please complete both columns for each item. | How often did you receive/seek out this type of support in the past week? | | | | | | | | Was this type of support available to you in the past week? | |
|  | No days (0) | On 1 day (1) | On 2 days (2) | On 3 days (3) | On 4 days (4) | On 5 days (5) | On 6 days (6) | Every day (7) | Yes (1) | No (0) |
| Emotional support (e.g., expressions of love, empathy, trust, caring) |  |  |  |  |  |  |  |  |  |  |
| Tangible (e.g., drive you to clinical placement, lend you money) |  |  |  |  |  |  |  |  |  |  |
| Informational (e.g., advice, suggestions, feedback) |  |  |  |  |  |  |  |  |  |  |
