# Supplementary material for: Individual differences and self-regulatory factors are credible determinants of physiotherapy student performance on clinical placement: Insights from a measurement burst design study
Source: Adv Health Sci Educ Theory Pract. 2025 Aug 4;31(2):573–606. doi: 10.1007/s10459-025-10453-4 (PMC13046584; doi:10.1007/s10459-025-10453-4)
Supplement: Supplementary file 3 — Supplementary Material 3: Supplementary Information File 3– Detailed statistical information [file 10459_2025_10453_MOESM3_ESM.docx]

***Sample size justification.*** Statistical power in multilevel models with multiple fixed and random effects is inherently complex, especially when prior studies do not offer reliable benchmarks for population effect sizes. Accordingly, our sample size planning was informed by practical considerations and estimates of the population size of Australian students undertaking placements in a fourth-year physiotherapy program. This approach is reasonable, given fourth-year physiotherapy students represent a small subset of both the general and student populations and thus a smaller number of participants is required to obtain a representative sample of this group (Lakens, 2022). Based on the data presented in Reubenson, Ng, Lawton et al. (2025), which analysed 8,979 clinical placement assessments from 1,865 graduating entry-level physiotherapy students across 19 Australian and New Zealand universities in 2019, we can estimate the representativeness of our sample as follows:

- Total Graduating Students in 2019: 1,865
- Our Sample (2019 Cohort): 141 students
- Estimated Representation: Approximately 7.6% of the 2019 graduating cohort

Assuming similar cohort sizes in 2020, our combined sample of 300 students over two years would represent roughly 16% of the total graduating physiotherapy students across Australia and New Zealand during that period. This proportion suggests that our sample is a meaningful subset of the national graduating cohort, supporting the generalisability of our findings within the context of Australian entry-level physiotherapy education.

Our decision to retain students who completed at least three surveys per burst for a minimum of two bursts was driven by our focus on intraindividual dynamics and the need for reliable estimation of within-person processes. As our research questions focus on how individual students’ experiences and perceptions fluctuate over time during their placements, repeated measures within individuals are essential. A minimum of three data points per burst allows us to obtain a rudimentary estimate of intraindividual variability, as fewer points would preclude the calculation of reliable and minimally biased estimates of within-person variance (e.g., Ram & Gerstorf, 2009; Rast et al., 2012). Also, the longitudinal aspect of our design necessitates observing individuals across multiple bursts to assess changes in these dynamics over time. Retaining only participants with at least two bursts enables us to examine within-person changes in variability and other dynamic processes across placement periods. We acknowledge that more data points would be ideal. Setting these minimum thresholds represents a pragmatic balance between maximising data quality, minimising participant burden, and retaining a reasonable sample size for analysis.

***Model Interpretation.*** We provide a brief section on model interpretation to assist readers who are unfamiliar with location-scale multilevel models. Each model encompasses fixed and random effects. Fixed effects represent the overall influence of a particular independent variable on the dependent variable *between-placements* and *between-persons*. Where fixed effects capture the average effect of certain factors that are assumed to be constant across all units at a given level, random effects capture deviations from this average. Said differently, random effects represent uncertainty in the data (e.g., due to measurement error, individual differences) unexplained by fixed effects. Given random effects are often considered nuisance parameters, we focus the remainder of our discussion on the interpretation of fixed effects.

Between-placement fixed effects indicate how levels of an independent variable from placement A to placement B predict an individual’s clinical or professional APP scores. Between-person fixed effects, on the other hand, indicate how an independent variable differentiates APP scores from person to person. We examined fixed effects for both mean levels of APP scores (location fixed effects) and the placement-to-placement variability of APP scores (scale fixed effects). Both location and scale fixed effects can be interpreted relative to their respective fixed intercepts. A fixed intercept refers to the value of the dependent variable when all independent variables in the model are zero. In our case, we centred independent variables at the cluster/placement mean (levels 2 and 3) or grand/sample mean (level 3). Doing so has the effect of making a model’s intercept equivalent to the APP score for a hypothetical “average” individual undertaking an “average” placement. Fixed effects represent how much mean levels or variability of APP scores increase/decrease when individuals report an independent variable as being one-unit above or below the average value for the sample or placement. In other words, fixed effects describe how scores shift as independent variable levels move away from the typical participant or placement.

Lakens, D. (2022). Sample Size Justification. *Collabra: Psychology*, *8*(1), 33267. https://doi.org/10.1525/collabra.33267

Ram, N., & Gerstorf, D. (2009). Time-structured and net intraindividual variability: tools for examining the development of dynamic characteristics and processes. *Psychology and aging*, *24*(4), 778.

Rast, P., MacDonald, S. W., & Hofer, S. M. (2012). Intensive measurement designs for research on aging. *GeroPsych, 25,* 45-55.

Reubenson A, Ng L, Lawton V, et al. (2025). The assessment of physiotherapy practice is a robust measure of entry-level physiotherapy standards: Reliability and validity evidence from a large, representative sample. PLoS ONE, 20(4): e0321397. <https://doi.org/10.1371/journal.pone.0321397>
